# MicroRNA‐7 deficiency ameliorates d‐galactose‐induced aging in mice by regulating senescence of Kupffer cells

**DOI:** 10.1111/acel.14145

**Published:** 2024-03-17

**Authors:** Ya Wang, Hui Qiu, Shipeng Chen, Dongmei Li, Xu Zhao, Mengmeng Guo, Nana Li, Chao Chen, Ming Qin, Ya Zhou, Daimin Xiao, Juanjuan Zhao, Lin Xu

**Affiliations:** ^1^ Department of Immunology Zunyi Medical University Zunyi Guizhou China; ^2^ Key Laboratory of Gene Detection and Treatment of Guizhou Province Zunyi Guizhou China; ^3^ School of Medicine Guizhou University Guiyang Guizhou China; ^4^ Department of Medical Physics Zunyi Medical University Zunyi Guizhou China; ^5^ Kweichow Moutai Hospital Renhuai Guizhou China

**Keywords:** aging, IL‐1β, KLF4, microRNA‐7, senescent Kupffer cells

## Abstract

Aging is intricately linked to immune system dysfunction. Recent studies have highlighted the biological function of microRNA‐7 (miR‐7) as a novel regulator of immune cell function and related diseases. However, the potential role of miR‐7 in aging remains unexplored. Here, we investigated the contribution of miR‐7 to d‐gal‐induced aging in mice, focusing on its regulation of senescent Kupffer cells. Our findings revealed that miR‐7 deficiency significantly ameliorated the aging process, characterized by enhanced CD4^+^ T‐cell activation. However, the adoptive transfer of miR‐7‐deficient CD4^+^T cells failed to improve the age‐related phenotype. Further analysis showed that miR‐7 deficiency significantly reduced IL‐1β production in liver tissue, and inhibiting IL‐1β in vivo slowed down the aging process in mice. Notably, IL‐1β is mainly produced by senescent Kupffer cells in the liver tissue of aging mice, and miR‐7 expression was significantly up‐regulated in these cells. Mechanistically, KLF4, a target of miR‐7, was down‐regulated in senescent Kupffer cells in aging mouse model. Furthermore, miR‐7 deficiency also modulated the NF‐κB activation and IL‐1β production in senescent Kupffer cells through KLF4. In conclusion, our findings unveil the role of miR‐7 in d‐gal‐induced aging in mice, highlighting its regulation of KLF4/NF‐κB/IL‐1β pathways in senescent Kupffer cells. This research may enhance our understanding of miRNA‐based aging immune cells and offer new avenues for new intervention strategies in aging process.

## Graphical abstract

It shows that miR‐7 deficiency ameliorates aging in mice by regulating senescent Kupffer cells. MiR‐7 deficiency reduces IL‐1β production in liver tissue, and inhibiting IL‐1β slows down the aging process. MiR‐7 regulates KLF4/NF‐κB/IL‐1β pathways in senescent Kupffer cells, contributing to the aging process.

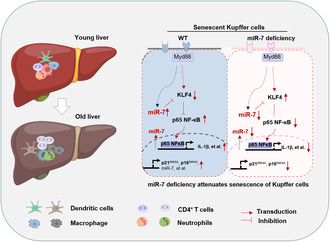

**Abbreviations**
d‐gal: d‐galactosamine
IL‐1β: interleukin‐1β
miR‐7: microRNA‐7
Nlrp3: Nod‐like receptor protein 3
ROS: reactive oxygen species
SASP: senescence associated secretory phenotype
TFAM: mitochondrial transcription factor A

## 1 INTRODUCTION

Aging is a complex process characterized by a decline in physiological function and an increased susceptibility to diseases (López‐Otín et al., 2023). Recent studies have identified several biological processes that serve as crucial markers of aging, including increased SA‐β‐gal activity, impaired antioxidant capacity, abnormal expression of cycle‐associated kinases, and a senescence associated secretory phenotype (SASP) (López‐Otín et al., 2023). These age‐related changes make individuals more susceptible to diseases, such as malignancies, diabetes, cardiovascular diseases, and neurodegenerative diseases, leading to higher mortality rates among the elderly (da Costa et al., 2016). Accumulating evidence has shown that aging is closely related to immune system dysfunction and immune‐related chronic inflammation. Meanwhile, the aged immune system can drive the senescence and aging of solid organs (Yousefzadeh et al., 2021). For instance, Minato et al. (2020) reported that T‐cell dysfunction provided a clue for controlling various age‐associated chronic inflammatory diseases and possibly cancer in humans. Moreover, macrophages in aged mice exhibited increased senescence markers (Wu et al., 2023). Serrano (2017) highlighted senescent cells in tissues as potential therapeutic targets for age‐associated diseases. However, the underlying mechanism of aging, especially different organ senescence, remains largely unknown, which is crucial for the development of therapeutic strategies against aging and aging‐related diseases in the future.

MicroRNA‐7 (miR‐7), a member of the miRNA family, has garnered attention for its role in the development of various mammalian diseases, including age‐related diseases. For instance, Jia et al. (2020) demonstrated that miR‐7 may have key roles in muscle aging. Furthermore, increased miR‐7 expression in elderly fibroblasts leads to decreased EGFR expression and a reduced TGF‐β1 response, thereby causing wound healing disorders (Midgley et al., 2014, 2016). Our recent series of studies have also indicated that miR‐7 is closely associated with inflammatory diseases (Zhao et al., 2016). For example, we found that the up‐regulation of miR‐7a‐5p, which targets RORα, was significantly linked to neuroinflammation in the pathogenesis of brain tissue inflammatory injury (Yue et al., 2020); moreover, we also shown that miR‐7 negatively controls CD4^+^ T‐cell activation and function through MAPK4, thereby orchestrating experimental autoimmune hepatitis in mice; additionally, we also demonstrated that miR‐7 served as a key intrinsic regulator in epithelial immunomodulation and regeneration, promoting colitis pathology (Zhao et al., 2023). However, the potential role of miR‐7 and its underlying mechanism in aging remain unclear.

In this study, we aimed to investigate the biological role of miR‐7 in aging and hypothesize that miR‐7 is an important regulator of aging. Our data revealed that miR‐7 deficiency significantly ameliorated the aging process in d‐gal‐induced aging mice. Although this improvement was accompanied by overactivation of CD4^+^T cells, miR‐7 deficient CD4^+^T cells did not improve the age‐related phenotype. Further analysis demonstrated that miR‐7 deficiency inhibited IL‐1β production in senescent Kupffer cells by regulating the KLF4/NF‐κB pathway, thereby improving d‐gal‐induced aging in mice. This not only enhances our understanding of the aging mechanism but also provides new targets for intervening in the aging process.

## 2 MATERIALS AND METHODS

### 2.1 Experimental animals and model

C57BL/6 wild‐type (WT) mice and miR‐7 deficiency (miR‐7^*def*^) mice (Male, 6–7 weeks) have been described in our previous study (Zhao et al., 2016, 2021, 2023). Rag1^−/−^ mice were purchased from Cyagen (Suzhou) Biotechnology Co., Ltd. Eighteen‐month‐old naturally aging male mice were purchased from Jiangsu ALF Biotechnology Co., LTD. The mice were housed under specific pathogen‐free (SPF) conditions at Zunyi Medical University, according to the guidelines for the Care and Use of Laboratory Animals (Ministry of Health, China, 1998). The experimental procedures were approved by the Zunyi Medical University Laboratory Animal Care and Use Committee (permit number SYXK‐2021‐00048).

*Murine Aging Model*: To induce the murine aging model, C57BL/6 WT mice (male, 6 weeks old) were subcutaneously injected with 300 mg/kg/day d‐gal (Sigma, G0750) for 8 weeks. The control group received an equal volume of PBS subcutaneously for 8 weeks.

Effect of IL‐1β neutralizing antibody on d‐gal‐induced aging: WT mice were subcutaneously injected with 300 mg/kg/day d‐gal for 8 consecutive weeks. IL‐1β neutralizing antibody (Bio X Cell, #BE0246) was intraperitoneally injected every other week (100 μg). The control group received intraperitoneal injections of an equal volume of PBS. After 8 weeks, the changes of age‐related indexes of mice were observed and analyzed.

### 2.2 Measurement of changes in weight index

The initial weight of each mouse was recorded before the experiment began. Subsequently, the weight of each mouse was recorded at specified time points after the experiment began. The weight index of each mouse was calculated using the following formula: weight index = current weight (g)/initial weight (g).

### 2.3 Histopathology

Liver, lung, and kidney tissues were fixed in 4% paraformaldehyde, embedded in paraffin, and cut into 3.5‐μm‐thick sections. Sections were stained with H&E, and images were taken with an Olympus IX71 microscope.

### 2.4 Biochemical analysis

The activity of SA‐β‐gal (U/g protein) (#BC2585) and SOD (U/g protein) (#BC1075), and the content of MDA (nmol/g protein) (#BC0025) in the liver, lung, and kidney tissues were measured by microplate reader with the assay kits purchased Beijing Solaybao Technology Co., Ltd. The content of ALT, AST, TC, TG, Cr, and Urea in serum was determined by the Second Affiliated Hospital of Zunyi Medical University.

### 2.5 Flow cytometry

The surface markers CD4‐Percp‐Cy5.5 (GK1.5, no. 12‐0041‐82), CD62L‐PE (MEL‐14, no. 12‐0621‐82), CD69‐APC (H1.2F3, no. 17‐0691‐82), CD8‐percp‐Cy7 (53‐6.7, no‐25‐0081‐82), CD44‐PE (IM7, 12‐0441‐82), CD25‐PE‐Cyanine7 (PC61.5, 25‐0251‐82), CD19‐PE (HIB19, no. 115508), Gr‐1‐FITC (RB6‐8C5, no. 108405), NK1.1‐percp‐Cy7 (PK136, no. 25‐5941‐82), CD11b‐PE (M1/70, no. 12‐0112‐82), CD11c‐APC (N418, no.MCD11c05), γδT‐APC (eBioGL3(GL3, GL3), no. 17‐5711‐81), F4/80‐Percp‐Cy5.5 (BM8, no. 45‐4801‐82), and transcriptional factors Ki67‐APC (SolA15, no. 17‐8698‐82) were evaluated by flow cytometry (FCM) with Beckman Gallios (Beckman Coulter, Inc.); for intracellular staining, cells were conducted according to the manufacturer's manual (eBioscience).

### 2.6 Fluorescence in situ hybridization

To evaluate the cellular distribution of miR‐7 in the liver tissue, a fluorescence in situ hybridization (FISH) assay was performed based on our previous description with some modifications (Yue et al., 2020). Briefly, before hybridization incubation, all solutions were prepared with diethylpyrocarbonate‐treated water. After deparaffinization and rehydration, tissue sections were treated by pepsin digestion. Sections were next incubated or heated in the microwave, and then were incubated with a hybridization cocktail containing a miR‐7 probe (5′‐ACAACAAAATCACTAGTCTTCCA‐3′, 1:250; EXIQON; no. 38485‐01) at 37°C overnight. The sections were washed in phosphate‐buffered saline (PBS) and incubated with a secondary antibody of Goat Anti‐Digoxigenin/Digoxin (DIG) Antibody, DyLight® 594 (1:250, DI‐7594 VECTOR) in the dark at room temperature for 1 h. The slides were rinsed with TBS‐T (0.5% Tween‐20) three times (5 min each) and counterstained, mounted with Slow Fade Gold Antifade Reagent with DAPI (1:1000) in the dark at room temperature for 10 min, and then examined by fluorescence microscopy.

### 2.7 Western blot

Western blot was performed on cytosolic cellular extracts. Equal amounts of protein were resolved under reducing conditions on a 10% SDS–polyacrylamide gel. Protein migration was assessed using protein standards (Bio‐Rad, CA). Transfer to a nitrocellulose membrane was performed for 1 h at 250 mA using a wet transfer system. Equal protein loading was confirmed with Ponceau staining. The membrane was washed in 5% skim milk in PBS plus 0.1% Tween 20 (PBST) for 1.5 h at 37°C to block nonspecific protein‐binding sites on the membrane. Immunoblotting has performed using a mAb to IL‐1β (Abcam, no. ab254360), p16^INK4A^ (Abcam, no. ab211542), p21^CIP1^ (Abcam, no. ab188224), KLF4 (Abcam, no. ab129473), NF‐κB (CST, no. 4764), p‐NF‐κB p65 (CST, no. 3039), and GAPDH (Abcam, no. ab9484) at a dilution of 1/1000 in a non‐fat milk‐Tris buffer. The membrane was then washed in TBS‐T and subsequently probed with a secondary anti‐mouse or rabbit Ab conjugated to HRP (CST, no. 7074) at a dilution of 1:5000. The signal was detected and analyzed using the Bio‐Rad ChemiDoc™ MP Imaging System (Bio‐Rad). Each experiment was performed in triplicate.

### 2.8 Cell culture and transfection

Mouse macrophage RAW264.7 cells (saved in our laboratory) were cultured in completed DMEM (GIBCO). RAW264.7 cells were transfected using Lipofectamine 3000 reagent with 100 nM of miR‐7 inhibitors, the inhibitors‐negative control (NC) and miR‐7 inhibitors+KLF4‐RNAi. The sequence of miR‐7 inhibitors is 5′‐AACAACAAAAUCACUAGUCUUCCA‐3′; the sequence of inhibitors‐NC is 5′‐CAGUACUUUUGUGUAGUACAA‐3′; KLF4 RNAi Sense: GGAGGGAGA‐CCGAGGAGUUTT, Anti‐sense: AACUCCUCGGUCUCCCUCCTT. Twelve hours later, these cells were treated with d‐gal (10 g/L) for 48 h. The cells were harvested at the indicated times.

*FITC‐Dextran uptake experiment*: RAW264.7 cells were transfected using Lipofectamine3000 reagent with 100 nM of miR‐7 inhibitors or the inhibitors‐negative control (NC). Twelve hours later, these cells were treated with d‐gal (10 g/L) for 48 h. Next, DMEM medium was used to adjust cell counts. Then, 3 × 10^5^ cells were added FITC‐Dextran (1 mg/mL) (FITC,MW10000,CAS No: 60842‐46‐8) and incubated at 37°C for 2 h. The cells were then washed with cold PBS at 1000 rpm for 10 min. Finally, the cells were suspended in 500 μL PBS for flow cytometry analysis.

### 2.9 Sorting of Kupffer cells

The hepatic portal vein was inserted with an indwelling needle and slowly irrigated with 20 mL D‐Hanks solution. The liver was excised and cut into a tissue block approximately 1 mm^3^, and the tissues were transferred to a 15 mL centrifuge tube and incubated in 2 mg/mL of type IV collagenase solution at 37°C for digestion. The digested tissues were then filtered through a cell strainer to remove any undigested tissue fragments. The cell suspension was centrifuged (500 *g*, 5 min), and the supernatant was discarded. The cell pellet was resuspended in Dulbecco's Modified Eagle's Medium (DMED) and centrifuged again. The supernatant was discarded, and the cell pellet was resuspended in phosphate‐buffered saline (PBS) and centrifuged. The supernatant was discarded, and the cell pellet was resuspended in 10% fetal bovine serum (FBS) in DMED. The cell suspension was counted using a hemocytometer, and 1 × 10^6^ cells were seeded onto each well of a six‐well plate. The cells were incubated at 37°C in a humidified atmosphere with 5% CO_2_ for 2 h. Non‐adherent cells were removed by gently washing the wells with PBS. The adherent cells were then collected and sorted as Kupffer cells.

### 2.10 Multiplexed fluorescent immunohistochemical staining

According to the Opal protocol of multiplexed fluorescent staining, slides were deparaffinized in xylene and rehydrated in ethanol. Antigen retrieval was performed in 1 mM Tris‐EDTA (pH 9.0) for 10 min using a 750‐W microwave and rinsed with TBS‐T three times, for 5 min each; the slides were blocked with 10% normal goat serum at room temperature for 10 min and then incubated with primary rabbit antibodies for p‐NF‐κB antibody (1:1000; CST; no. 3033) overnight at 4°C. After the overnight incubation, the slides were rinsed with TBS‐T three times and incubated with Goat anti‐Rabbit IgG H&L (HRP) secondary antibody (1:1000 dilution; Abcam; no. ab6721) at room temperature for 1 h. Later, phos‐NF‐κB was visualized using PPD520 tyramine signal amplification Plus (1:100). Subsequently, the slides were placed in Tris‐EDTA (pH 9.0), subjected to microwave again, and then incubated with primary rabbit antibodies for KLF4 (1:1000; CST, no. 4038) in a humidified chamber at room temperature for 2 h. After incubating with Goat Anti‐Rabbit IgG H&L (HRP) secondary antibody for 1 h, KLF4 was visualized using PPD650 tyramine signal amplification. After washing in TBS‐T three times, sections were counterstained, mounted with Slow Fade Gold Antifade Reagent with DAPI, and left for 10 min in the dark at room temperature before examination by fluorescence microscopy.

### 2.11 Immunofluorescence (IF)

Sections were hydrated and rinsed with PBS three times (5 min each) and then blocked with 10% normal goat serum at room temperature for 10 min and incubated with rabbit anti‐mouse antibodies at appropriate dilution in TBS overnight at 4°C. The primary antibodies used were as follows: CD68 (1:100; Abcam, no. ab283654), albumin (1:400; Abcam; no. ab207327), and Ki67 (1:500; GeneTex, no. GTX16667). PBS instead of primary antibody served as a control. Then, the slices were rinsed with cold PBS three times (5 min each) and incubated with the secondary antibody of Alexa Fluor 488‐conjugated Goat Anti‐Rabbit IgG (1:500; Invitrogen) and Alexa Fluor 647‐labeled Goat Anti‐Rabbit IgG (1:500; CST) in the dark at room temperature for 1 h. Finally, the sections were mounted with Slow Fade Gold Antifade Reagent with DAPI and examined by fluorescence microscopy.

### 2.12 Real‐time PCR

The conventional primers were obtained from Shanghai Sangon Biological Engineering CO. The TaqMan probes of MiR‐7 (000386) and U6 (001793) were purchased from Life Technologies; the other reagents were from TAKARA Bio Inc. Reverse transcriptase reactions and real‐time PCR assays were performed according to the manufacturer's protocols. All reverse transcriptase reactions, including no‐template controls and reverse transcriptase minus controls, were run in triplicate in BIO‐RAD CFX96 (Bio‐Rad Laboratories). The expression of miR‐7 was performed according to the TaqMan assays, and samples were normalized by evaluating U6 expression. The other following gene‐expression levels were quantified using the BIO‐RAD CFX96 detection system (Bio‐Rad Laboratories), and the samples were normalized by evaluating GAPDH. Relative expression was calculated using the comparative threshold cycle (*C*_t_) method. In the experimental results presented in Figures 3a and 5b and Figure S5A, showing the fold increase or decrease after comparison are presented. The fold change of up‐regulation or down‐regulation of one group was calculated by normalizing the other group to 1. This was done by dividing the expression level of the up‐regulated or down‐regulated group by the mean expression level of the control group. The primers used are listed in Table S1.

### 2.13 Statistical analyses

The data were analyzed with GraphPad Prism 7.0 and are presented as the mean ± SD. Student's unpaired *t* test was used when two conditions were compared, and analysis of variance with Bonferroni or Newman–Keuls correction was used for multiple comparisons. *p*‐Values of <0.05 were considered to be significant.

## 3 RESULTS

### 3.1 MiR‐7 deficiency ameliorates aging in d‐gal induced murine model

To investigate the potential role of miR‐7 in aging, we constructed a d‐gal‐induced murine aging model (Figure S1A). Compared to the WT mice in the PBS group, there were significant changes in age‐related indicators in the WT mice in the d‐gal group. These changes were reflected in the following: a significant decrease in the weight index and organ index of the liver and kidney, a significant increase in the lung organ index, and a significant increase in levels of ALT, AST, TG, TC, Urea, and Cr in the serum (Figure S1B–D). Extensive inflammatory damage was observed in the liver, lung, and kidney tissues (Figure S1E), accompanied by a significant up‐regulation of the expression of the pro‐inflammatory factor *IL‐6* and a significant down‐regulation of the expression of the anti‐inflammatory factor *IL‐10* (Figure S1F). SA‐β‐gal activity and MDA content were significantly increased in the liver, lung, and kidney tissues, while SOD activity was significantly decreased (Figure S1G–I). These data indicated that the d‐gal induced murine aging model was successfully constructed.

**FIGURE 1:**
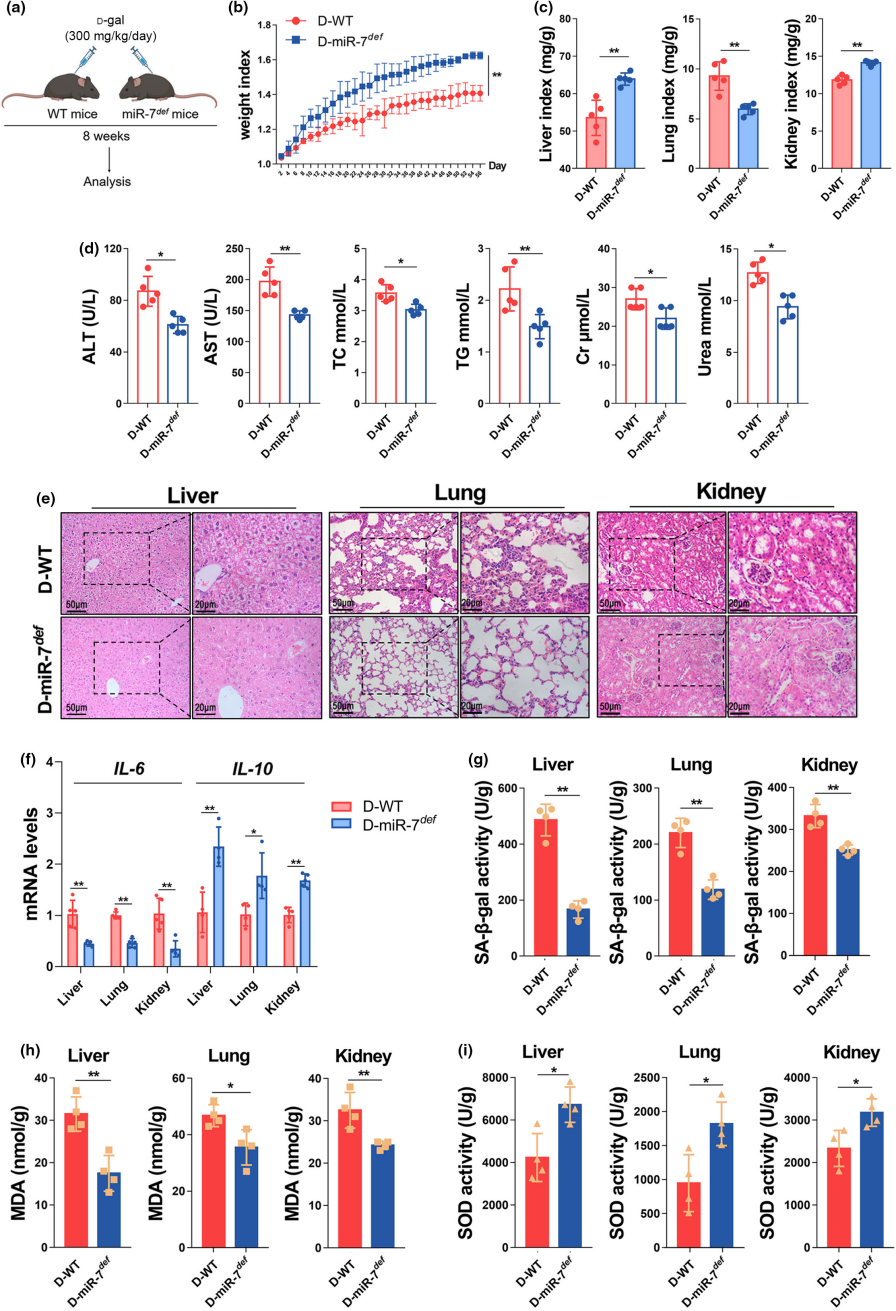
MiR‐7 deficiency alleviates murine aging induced by d‐gal. (a) The schematic representation of the experiments of murine d‐gal‐induced aging. MiR‐7^*def*^ mice and WT mice (6–7 weeks old, *n* = 5) were given 300 mg/kg/day d‐gal solution for 8 weeks, and the mice were sacrificed on the 56th day. The weight index (b) and organ index (c) of WT mice and miR‐7^*def*^ mice after d‐gal treatment were observed; the concentration of the serum ALT, AST, TC, TG, Cr, and Urea was detected and analyzed (d). Histopathology of the liver, lung, and kidney tissue was performed by H&E staining (left, 50 μm; right, 20 μm (e)). The relative expression levels of cytokines *IL‐6* and *IL‐10* were assessed in the liver, lung, and kidney tissues by real‐time PCR assay (f). The activity of SA‐β‐gal (g) and SOD (i), and the content of MDA (h) in the liver, lung, and kidney were detected by biochemical reagent kit. The values are the means ± SD (*n* = 5). *p < 0.05, **p < 0.01.

To observe the potential role of miR‐7 in aging, we induced miR‐7 deficient mice with d‐gal (Figure 1a). Compared to the WT mice in the d‐gal group, the weight and organ indexes (the liver, lung, and kidney tissues) were significantly recovered in the miR‐7 deficient mice in the d‐gal group (Figure 1b,c), and the function of major organs was significantly improved, including the reduced levels of ALT, AST, TC, TG, Cr, and Urea in the serum (Figure 1d). H&E staining further showed a significant reduction in cell edema in the liver tissue, no significant widening of the alveolar interval in lung tissue, and no significant atrophy of the glomerulus in kidney tissue (Figure 1e). This was accompanied by a significant down‐regulation of the expression of the pro‐inflammatory factor *IL‐6* and significant up‐regulation of the expression of the anti‐inflammatory factor *IL‐10* (Figure 1f). Importantly, SA‐β‐gal activity and MDA content in the liver, lung, and kidney tissues of D‐miR‐7^*def*^ mice were also significantly decreased, while SOD activity was significantly increased (Figure 1g–i). These results suggest that miR‐7 deficiency significantly ameliorated the aging process in d‐gal‐induced murine aging.

### 3.2 MiR‐7 deficiency does not improve aging by affecting CD4 ^+^T cells

In light of our previous work showing that miR‐7 plays an important regulatory role in immune organ development and multiple immune cell functions (Chen et al., 2021; Hu et al., 2021; Zhao et al., 2021). Therefore, we investigated whether miR‐7 affects the immune system and immune cells in aging mice. We observed significantly obstructed development and differentiation of T cells in the thymus (Figure S2A–C) and significantly reduced proportions and absolute numbers of multiple immune cell subsets in the splenocytes in D‐WT mice compared to the P‐WT group (Figure S2E–H). Compared to mice in the D‐WT group, the thymus volume and total number of cells in D‐miR‐7^*def*^ mice were significantly restored, and the development and differentiation of T cells were significantly improved (Figure S3A–D). The splenic volume and total number of immune cells increased significantly, and the proportion and number of some immune cell subsets recovered significantly, especially the absolute number of CD4^+^T cells, which was about 4 times higher (Figure S3E–G).

**FIGURE 2:**
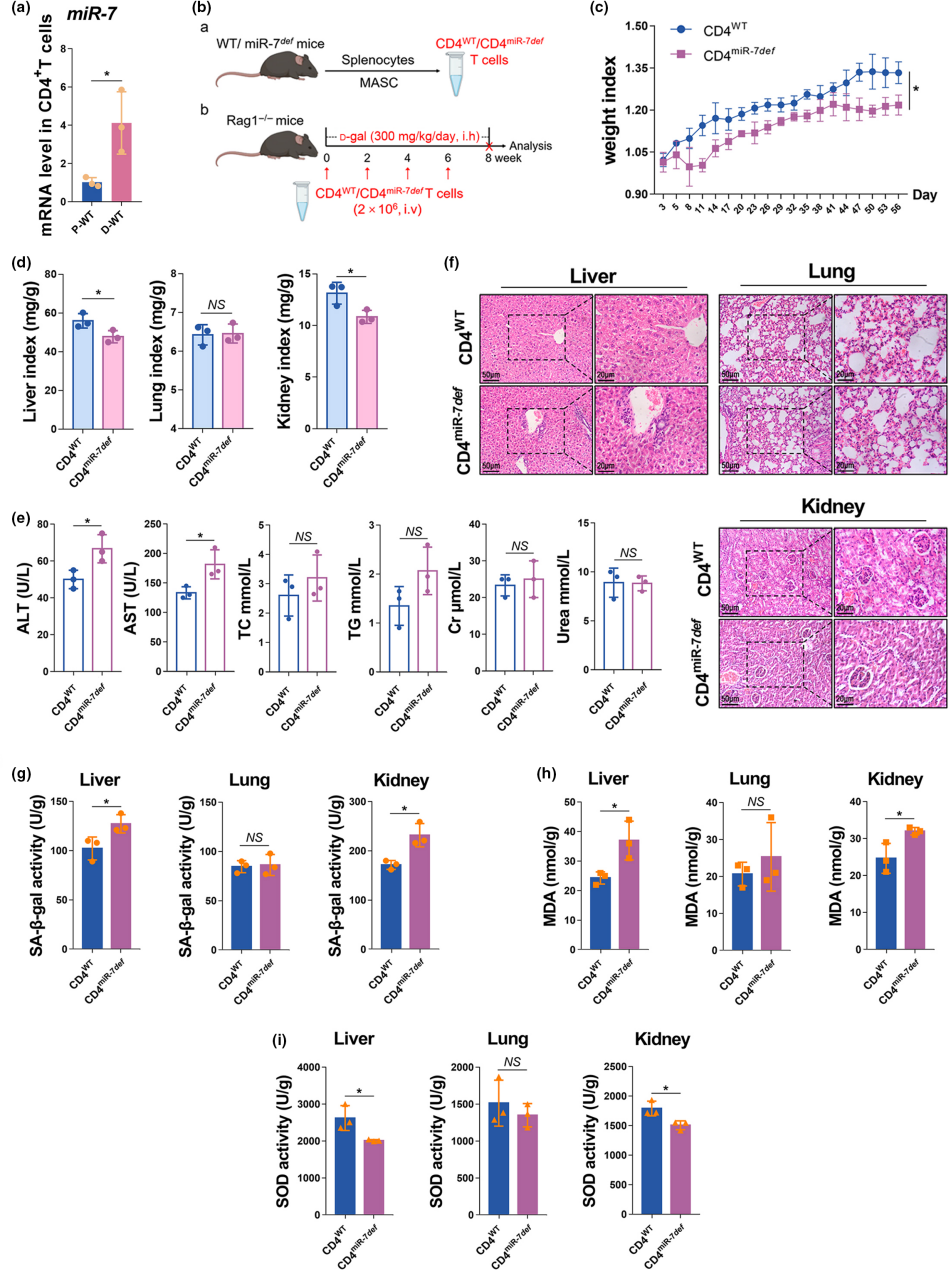
MiR‐7 does not improve murine aging by affecting CD4^+^T cells. (a) The level of miR‐7 in CD4^+^T cells from the splenocytes of PBS treated WT and d‐gal treated WT mice was detected by real‐time PCR. (b) The schematic representation of the generation of the adoptive transfer assay. CD4^+^T cells purified by MACS in splenocytes from WT and miR‐7^*def*^ mice (6–7 weeks, *n* = 3) were transferred into syngeneic Rag1^−/−^ mice(6 weeks) through tail vein injection every 2 weeks, and these mice were administered 300 mg/kg d‐gal (i.h.) for 8 weeks. The weight index (c) and organ index (d) were observed on the 56th day. The concentration of the serum ALT, AST, TC, TG, Cr, and Urea was detected and analyzed (e). Histopathology of the liver, lung, and kidney was performed by H&E staining (left, 50 μm; right, 20 μm) (f, g). The activity of SA‐β‐gal (h) and SOD (i), and the content of MDA (i) in the liver, lung, and kidney were detected by biochemical reagent kit. The values are the means ± SD (*n* = 5). *p < 0.05.

Recent studies have revealed the key role of CD4^+^T cells in aging (Hasegawa et al., 2023; Zhang et al., 2023). Therefore, we further examined the expression level of miR‐7 in splenic CD4^+^T cells of D‐WT mice. The results showed that a significant up‐regulation of miR‐7 expression in CD4^+^T cells (Figure 2a). Next, we further analyzed the changes of CD4^+^T cells in the splenocytes of D‐miR‐7^*def*^ mice. As expected, the proportion of CD4^+^CD69^+^T cells and CD4^+^Ki67^+^T cells increased significantly, while the proportion of CD4^+^CD62L^+^T cells decreased significantly (Figure S3H). These results illustrated that CD4^+^T‐cell activation and proliferation were enhanced in the splenocytes of miR‐7^*def*^ mice induced by d‐gal, which was consistent with our previous findings (Zhao et al., 2021). Collectively, these data suggested that miR‐7 deficiency may ameliorate murine aging mainly by affecting CD4^+^T cells.

To further explore the possible role of miR‐7‐deficient CD4^+^T cells in aging process, we adopted transfer CD4^WT^ T cells and CD4^miR‐7*def*^ T cells to Rag1^−/−^ mice via the tail vein, respectively, and then induced by d‐gal (Figure 2b). After the adoptive transfer of miR‐7 deficient CD4^+^T cells, the number of immune cells and the activation of CD4^+^T cells in the murine splenocytes were significantly increased (Figure S4A–C). The weight indexes, the liver and kidney indexes of mice transfused with CD4^miR‐7*def*^ T cells were significantly reduced (Figure 2c,d). The liver function of mice was significantly impaired, including a significant increase in the serum ALT and AST levels (Figure 2e), aggravated pathological damage, and significant increase in the infiltrated inflammatory cells (Figure 2f,g). SA‐β‐gal activity and MDA content in the liver and kidney tissues of mice were significantly increased, while SOD activity was significantly decreased (Figure 2h–j). Although there was no significant difference in the changes of relevant indexes in the lung tissues of the two groups of mice, both showed a trend of aging‐related changes (Figure 2i,j). Together, these results revealed that the adoptive transfer of CD4^miR‐7*def*^ T cells does not improve murine aging, but rather promotes age‐related phenotypes to some extent, which suggested that miR‐7 deficiency does not improve d‐gal induced aging in mice by affecting CD4^+^T cells.

### 3.3 Neutralization of IL‐1β significantly improves murine aging in vivo

**FIGURE 3:**
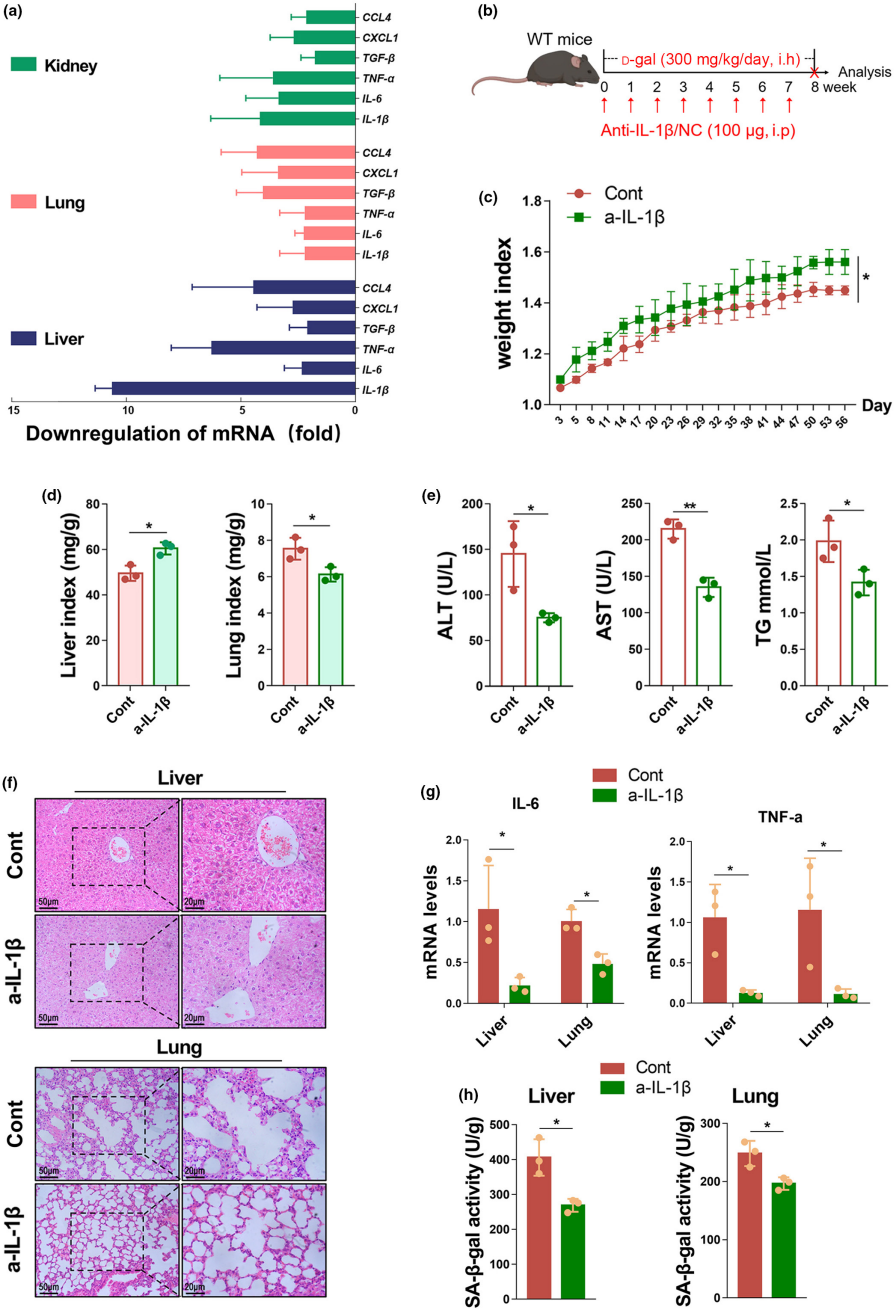
Neutralizing IL‐1β in vivo can improve murine aging induced by d‐gal. (a) The relative expression levels of proinflammatory cytokines *IL‐1β*, *IL‐6*, *TNF‐α*, *TGF‐β*, *CXCL1*, and *CCL4* were assessed in the liver, lung, and kidney tissue from WT mice and miR‐7^*def*^ mice after d‐gal treatment by real‐time PCR assay. (b) A schematic representation of the neutralizing IL‐1β in vivo experiment is shown. WT mice (6–7 weeks old, *n* = 3) were given 300 mg/kg/day d‐gal solution for 8 weeks, while intraperitoneal injection of anti‐IL‐1β was given every other week to neutralize IL‐1β in vivo; the mice were sacrificed on the 56th day. The weight index (c) and organ index (d) of the WT mice with or without anti‐IL‐1β treatment were observed. The concentration of the serum ALT, AST, and TG was detected and analyzed (e). Histopathology of liver and lung was performed by H&E staining (left, 50 μm; right, 20 μm) (f). The relative expression levels of *IL‐6* and *TNF‐α* were assessed in the liver and lung tissue by real‐time PCR assay (g). The activity of SA‐β‐gal in liver and lung was detected by a biochemical reagent kit (h). The values are the means ± SD (*n* = 5). *p < 0.05, **p < 0.01.

It is worth mentioning that our previous series of studies have shown that miR‐7 plays an important regulatory role in various inflammatory diseases and is accompanied by differential expression changes of various inflammatory factors (Chen et al., 2021; Zhao et al., 2016, 2021, 2023). In this study, we also found significant changes in inflammatory factors in the organs including the liver, lung, and kidney tissues, due to miR‐7 deficiency (Figure 1f). Studies have shown that inflammatory factors accelerate the aging process (Campeau et al., 2022; Panza et al., 2015; Villeda et al., 2011). Based on these data, we hypothesized that the inflammatory factors regulated by miR‐7 may be involved in the aging process. Therefore, we further analyzed the expression of aging‐related inflammatory factors in the organs including the liver, lung, and kidney tissues of aging mice with miR‐7 deficiency. The results showed the following: compared with the P‐WT group, the expression levels of *IL‐1β*, *TNF‐α*, *IL‐6*, *TGF‐β*, *CXCL1*, and *CCL4* in the liver, lung, and kidney tissues of the D‐WT group were significantly up‐regulated, with the *IL‐1β* level in liver tissues being up‐regulated by about 13‐flod (Figure S5A). Compared with the D‐WT group, the age‐related inflammatory factors in the liver tissues of D‐miR‐7^*def*^ mice were most significantly down‐regulated, including *IL‐1β*, *TNF‐α*, *IL‐6*, *TGF‐β*, *CXCL1*, and *CCL4*, with the *IL‐1β* level being most significantly down‐regulated by about 10‐fold (Figure 3a). These results suggest that IL‐1β may play an important role in murine aging.

To confirm the role of IL‐1β in aging, we conducted in vivo neutralization of IL‐1β experiments on aging mice (Figure 3b). The results showed that, compared with the Control (Cont) group, the weight and organs indexes of mice in the anti‐IL‐1β (a‐IL‐1β) group significantly recovered (Figure 3c,d). The liver function injury was significantly reduced, with a significant decrease in serum ALT, AST, and TG levels (Figure 3e); however, there was no statistical difference in serum TC, Urea, and Cr levels between the two groups (Figure S6A). H&E staining further demonstrated a significant reduction in the pathological damage in liver, lung, and kidney tissues, with only a small number of inflammatory cells infiltrating (Figure 3f, Figure S6B). Consistently, the levels of age‐related inflammatory factors *IL‐6* and *TNF‐α* were significantly down‐regulated (Figure 3g). Importantly, the aging‐associated phenotypes, including SA‐β‐gal and *p21* ^*CIP1*^ and *p16* ^*INK4A*^ expression levels, were significantly reduced in liver and lung tissues, with the most significant reduction observed in the liver tissues, although there was no significant change in kidney tissues (Figure 3h, Figure S6C,D). These findings provide strong evidence that IL‐1β plays a critical role in aging and that neutralization of IL‐1β can ameliorate age‐related decline and extend lifespan in mice.

### 3.4 MiR‐7 mainly regulates the production of IL‐1β in senescent Kupffer cells

**FIGURE 4:**
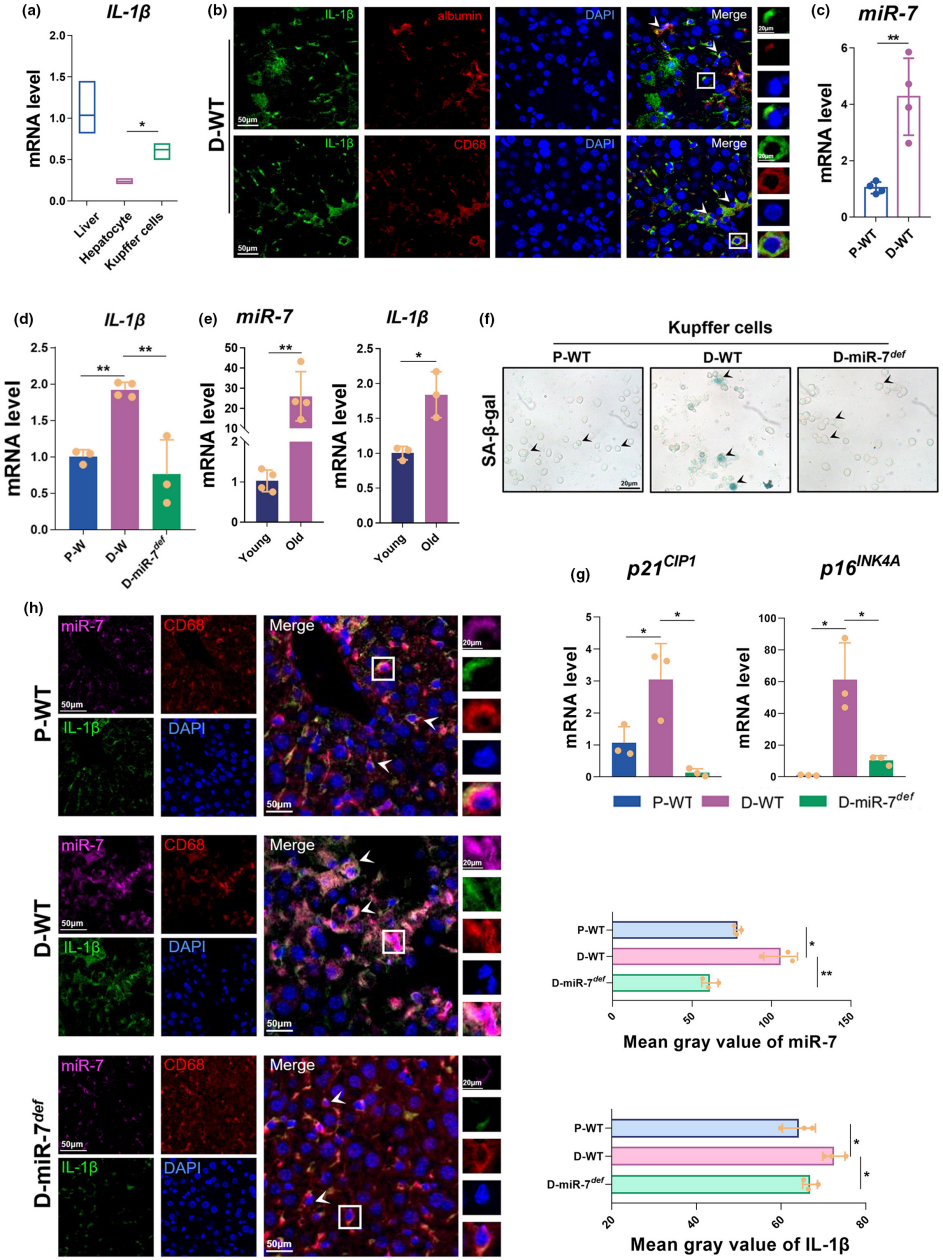
IL‐1β in the liver of aging mice is mainly produced by Kupffer cells. (a) The relative expression level of *IL‐1β* was assessed in the liver tissue, hepatocytes, and Kupffer cells by real‐time PCR assay. (b) The expression of IL‐1β, CD68, and albumin was determined by multiplexed fluorescent immunohistochemical staining in the liver (left, 50 μm; right, 20 μm). (c) The expression of miR‐7 was assessed in the Kupffer cells from P‐WT and D‐WT mice by real‐time PCR assay. (d) The expression of *IL‐1β* was assessed in the Kupffer cells from P‐WT, D‐WT, and D‐miR‐7^*def*^ mice by real‐time PCR assay. (e) The expression of miR‐7 and *IL‐1β* was assessed in the Kupffer cells from 2‐month‐old (young) and 18‐month‐old (old) mice by real‐time PCR assay. (f) SA‐β‐Gal staining of Kupffer cells from liver of WT mice after subcutaneous injection of d‐gal. (g) The relative expression levels of *p21* ^*CIP1*^ and *p16* ^*INK4A*^ were assessed in the Kupffer cells from P‐WT, D‐WT and D‐miR‐7^*def*^ mice by real‐time PCR assay. (h) The expression of miR‐7, CD68, and IL‐1β was determined by FISH in the liver (left, 50 μm; right, 20 μm). The values are the means ± SD (*n* = 5). *p < 0.05, **p < 0.01.

Next, we further monitored the major producing tissues and cells of IL‐1β production and its correlation with miR‐7 expression in aging mice. Our previous data showed that changes in IL‐1β expression were most significant in liver tissue. Therefore, we took the liver as the representative and first detected the expression and localization of IL‐1β in primary Kupffer cells and hepatocytes in the liver of D‐WT mice using real‐time PCR and immunofluorescence. The results showed that the expression level of *IL‐1β* in Kupffer cells of D‐WT mice was significantly higher than that in hepatocytes and accounted for more than 50% of the expression level of *IL‐1β* in liver tissues (Figure 4a). Immunofluorescence analysis also revealed that IL‐1β was primarily co‐localized with CD68^+^ Kupffer cells in the liver (Figure S5B, Figure 4b). Next, we examined the miR‐7 expression in Kupffer cells in aging mice. Real‐time PCR assay results showed that the expression of miR‐7 in Kupffer cells of D‐WT mice was significantly up‐regulated, and the level of *IL‐1β* was also significantly increased (Figure 4c,d). Similarly, compared with 2‐month‐old young mice, the expression of miR‐7 and *IL‐1β* in Kupffer cells of 18‐month‐old naturally aged mice was significantly increased (Figure 4e). In addition, Kupffer cells displayed a distinct age‐related phenotype, with increased expression of SA‐β‐gal, up‐regulation of *p21* ^*CIP1*^ and *p16* ^*INK4A*^ expression levels (Figure 4f,g), and significantly reduced cell proliferation (Figure S5C). After miR‐7 deficiency, the expression level of IL‐1β in Kupffer cells was significantly down‐regulated, and the age‐related phenotype was significantly improved (Figure 4f,g). These results were further confirmed by FISH assay (Figure 4h). Combining these data, we conclude that miR‐7 mainly regulates the production of IL‐1β in senescent Kupffer cells.

### 3.5 MiR‐7 regulates IL‐1β production by regulating the KLF4/NF‐κB pathway in Kupffer cells

**FIGURE 5:**
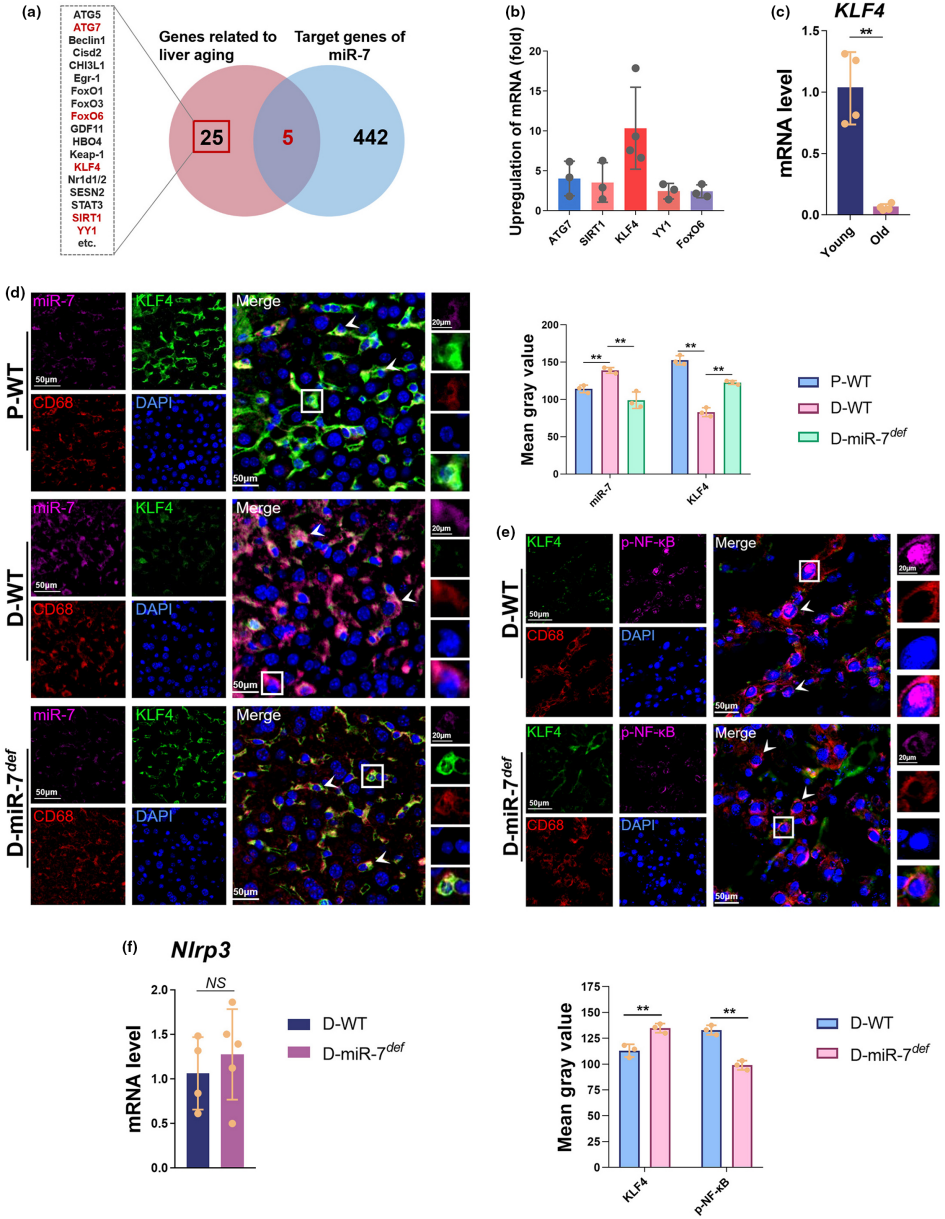
KLF4 is a target of miR‐7 in Kupffer cells. (a) Wenny analysis of miR‐7 target genes and related literature resulted in the screening out of five genes that are potentially interesting. (b) Real‐time PCR analysis for *ATG7*, *SIRT1*, *KLF4*, *YY1*, and *FoxO6* was performed in murine Kupffer cells. (c) The expression of *KLF4* was assessed in Kupffer cells from 2‐month‐old (young) and 18‐month‐old (old) mice by real‐time PCR assay. (d) The expression of miR‐7, KLF4, and CD68 was determined by FISH assay in the liver from P‐WT, D‐WT, and D‐miR‐7^*def*^ mice (left, 50 μm; right, 20 μm). (e) The expression of KLF4, p‐NF‐κB, and CD68 was determined by multiplexed fluorescent immunohistochemical staining in the liver (left, 50 μm; right, 20 μm). (f) The expression of *Nlrp3* was assessed in Kupffer cells from D‐WT and D‐miR‐7^*def*^ mice by real‐time PCR assay. The values are the means ± SD (*n* = 5). **p < 0.01.

To further investigate the mechanisms through which miR‐7 regulates IL‐1β production in senescent Kupffer cells, we utilized TargetScan databases (https://www.targetscan.org/) and combined them with existing studies, to identify five potential target genes *ATG7* (Guo et al., 2022), *SIRT1* (Maeso‐Díaz et al., 2018), *KLF4* (Ji et al., 2019), *YY1* (Tang et al., 2020), and *FoxO6* (Kim et al., 2019) of miR‐7 that are associated with liver tissue aging (Figure 5a). We then examined the expression of these potential target genes in Kupffer cells from D‐miR‐7^*def*^ mice and found that *KLF4* showed the most significant enrichment among the five genes in Kupffer cells (Figure 5b). Similarly, the expression of *KLF4* in Kupffer cells form 18‐month‐old naturally aged mice was significantly down‐regulated compared to that in Kupffer cells from 2‐month‐old young mice (Figure 5c), but the expression of miR‐7 has a significant positive correlation with age (Figure 4e). Furthermore, FISH results also demonstrated a negative correlation between miR‐7 expression and KLF4 expression (Figure 5d). More importantly, KLF4 and miR‐7 were dominantly co‐localized in Kupffer cells (Figure 5d). These findings suggest that KLF4 is an important target molecule for miR‐7 in senescent Kupffer cells induced by d‐gal.

It has been reported that the production of IL‐1β is regulated the NF‐κB/Nlrp3 inflammasome pathway (Enzan et al., 2023). Therefore, we further analyzed the effects of miR‐7 deficiency on NF‐κB activation and Nlrp3 expression. Immunofluorescence results revealed that the expression of p‐NF‐κB was significantly increased in Kupffer cells from D‐WT mice, along with a decrease in KLF4 expression (Figure 5e). Furthermore, Kupffer cells from D‐miR‐7^*def*^ mice displayed significantly increased KLF4 expression and decreased p‐NF‐κB expression (Figure 5e). Surprisingly, real‐time PCR results showed no significant difference in *Nlrp3* expression in liver Kupffer cells from the D‐miR‐7^*def*^ group and the D‐WT group (Figure 5f). These data suggest that KLF4, one of the targets of miR‐7, targets to regulate NF‐κB activation and subsequently IL‐1β production in Kupffer cells of d‐gal‐induced aging mice.

### 3.6 MiR‐7 influences macrophage senescence through the regulation of the KLF4/NF‐κB/IL‐1β pathway

Studies have reported that aged immune cells drive senescence and aging of solid organs (Yousefzadeh et al., 2021). Hence, we further validated the correlation between of miR‐7/KLF4 axis and Kupffer cell senescence. We induced senescence in murine macrophage RAW264.7 cells in vitro using d‐gal. As expected, the number of β‐gal^+^ cells in d‐gal‐induced macrophages was significantly increased in the d‐gal group (Figure S7A). Additionally, there was a significant up‐regulation of p16^INK4A^ and p21^CIP1^ expression (Figure S7B,E), accompanied by reduced cell proliferation (Figure S7C). Furthermore, the expression of miR‐7 and IL‐1β was significantly increased, while KLF4 expression was significantly down‐regulated (Figures S7D,E and S8).

**FIGURE 6:**
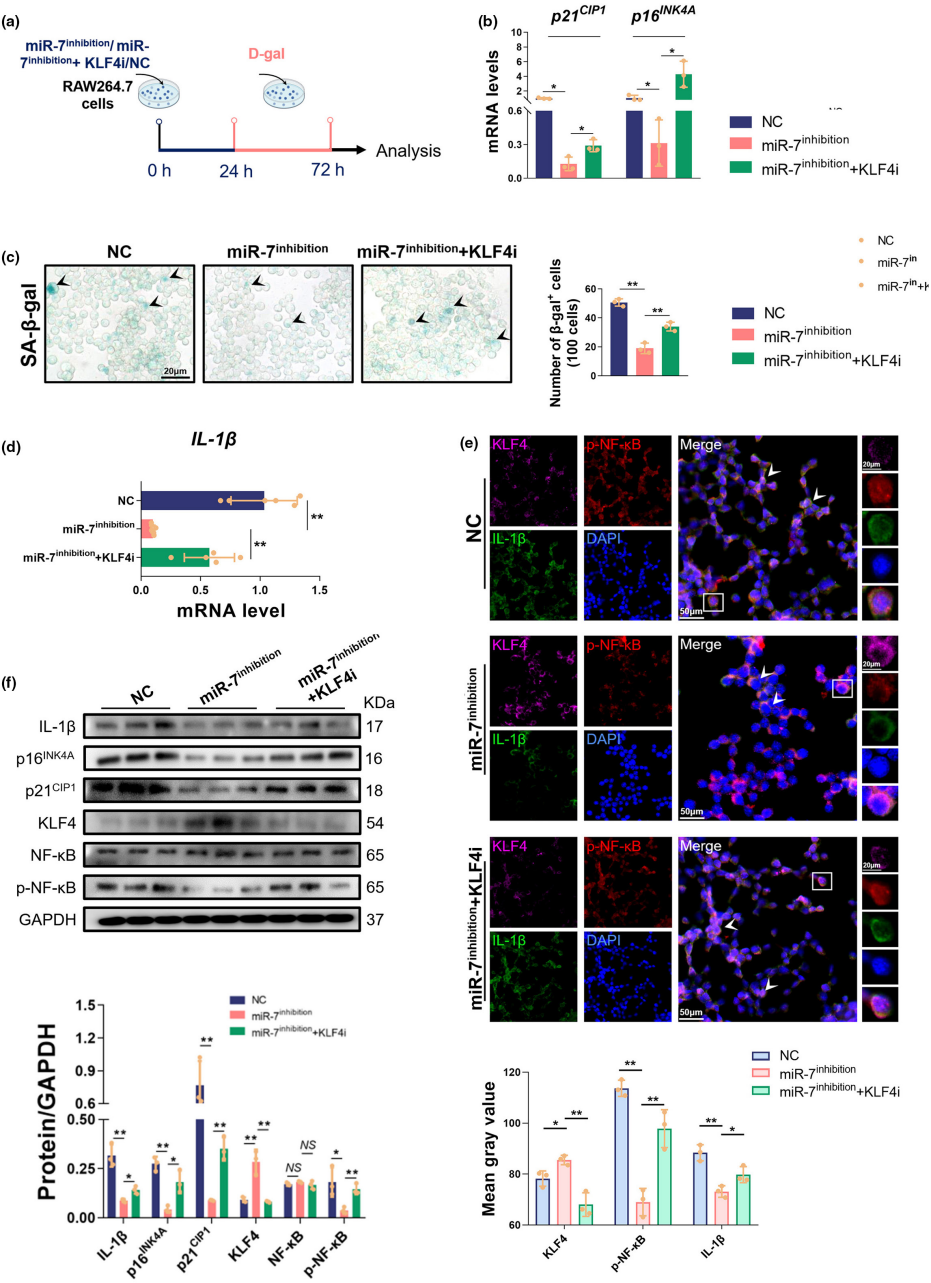
MiR‐7 controls the senescence of macrophages via KLF4/NF‐κB/IL‐1β pathway in vitro. (a) Murine macrophages RAW264.7 cells were treated with NC/miR‐7 inhibitors/miR‐7 inhibitors+KLF4‐RNAi (100 nM); 24 h later, these cells were continuously treated with d‐gal for 48 h. (b) Real‐time PCR analysis for *p21* ^*CIP1*^ and *p16* ^*INK4A*^ in these cells. (c) SA‐β‐gal staining for RAW264.7 cells with NC/miR‐7 inhibitors/miR‐7 inhibitors +KLF4‐RNAi. (d) The expression of *IL‐1β* was assessed in these cells by real‐time PCR assay. (e) The expression of KLF4, p‐NF‐κB, and IL‐1β was determined by multiplexed fluorescent immunohistochemical staining in the cells of each group (left, 50 μm; right, 20 μm). (f) Immunoblot analysis of IL‐1β, p16^INK4A^, p21^CIP1^, KLF4, NF‐κB, and p‐NF‐κB in the cells of each group. The values are the means ± SD (*n* = 5). *p < 0.05, **p < 0.01.

Subsequently, we examined the potential effects of the miR‐7/KLF4/NF‐κB/IL‐1β axis on senescent macrophages. We first silenced miR‐7 expression using a miR‐7 inhibitor to observe the possible changes in aging‐related indicators (Figure 6a). After treatment with the miR‐7 inhibitor, the expressions of *p16* ^*INK4A*^ and *p21* ^*CIP1*^ were significantly down‐regulated (Figure 6b), the number of β‐gal^+^ cells significantly decreased (Figure 6c), and, interestingly, the phagocytic and proliferative function of senescent macrophages was enhanced (Figures S9 and S10). Simultaneously, IL‐1β production was significantly reduced, and the expression of p‐NF‐κB was significantly down‐regulated (Figure 6e,f, Figure S8). Notably, treatment with a KLF4 inhibitor could also reverse the effect of miR‐7 deficiency on senescent macrophages, including a diminished proliferative capacity and up‐regulation of p16^INK4A^ and p21^CIP1^ expression, a significant increase in IL‐1β production (Figure S10, Figure 6d), and a significant up‐regulation in p‐NF‐κB expression (Figure 6e). Overall, these data suggest that miR‐7 regulates IL‐1β production by controlling the KLF4/NF‐κB pathway, consequently influencing macrophage senescence.

## 4 DISCUSSION

Recently, accumulating evidence has shown that miR‐7 plays a promising role in regulating the biological processes of various diseases, and has become a potential new target for clinical disease intervention (Zhao et al., 2021, 2023). Our prior research series has uncovered that miR‐7 also regulates the pathologies of autoimmune hepatitis (Zhao et al., 2021), inflammatory bowel disease (Zhao et al., 2023), acute lung injury (Zhao et al., 2016), and brain tissue inflammatory injury (Yue et al., 2020). In this study, we extended previous findings by demonstrating that miR‐7 deficiency significantly slows aging in a d‐gal‐induced mouse model. This was reflected in the recovery of weight and organ index, improvement in main organ function, tissue structure and antioxidant capacity, decrease in pro‐inflammatory factor expression and β‐galactosidase activity in tissues. These observations suggest a crucial regulatory role of miR‐7 in aging and related pathological processes. Concordant with our findings, other researchers have reported high levels of miR‐7 in the skeletal muscle of older mice and deer, and miR‐7 expression is inversely correlated with muscle health in mice and humans (Jia et al., 2020; Yang et al., 2022). Furthermore, non‐coding RNA molecules such as miR‐34 (Srinivasan et al., 2022), miR‐188‐3p (He et al., 2022), and miR‐21 (Xiong et al., 2021) have been implicated in the regulation of the aging process. Collectively, these studies highlight the critical regulatory role of non‐coding RNA molecules, represented by miR‐7, in the aging process. This knowledge provides a solid foundation for the development of novel therapeutic targets for future interventions aimed at modulating aging‐related pathologies.

Accumulating evidence suggests the involvement of CD4^+^ T cells, key adaptive immune cells, in various clinical diseases and the aging process. Our previous studies demonstrated that miR‐7 deficiency led to overactivation of CD4^+^T cells and aggravated the pathological damage of autoimmune hepatitis (Zhao et al., 2021). In this study, we observed that the expression of miR‐7 was significantly up‐regulated in splenic CD4^+^T cells of aging mice, and the proliferation and activation of CD4^+^T cells significantly increased after miR‐7 deficiency. However, adoptive transfer of miR‐7‐deficient CD4^+^T cells failed to improve murine aging and, instead, promoted age‐related phenotypes. Similarly, Desdín‐Micó et al. (Desdín‐Micó et al., 2020) reported that CD4^+^T cells with dysfunctional mitochondria due to mitochondrial transcription factor A (TFAM) deficiency produced high levels of inflammatory cytokines *IL‐6*, TNF‐α, etc., which act as accelerators of senescence. Muyayalo et al. (Muyayalo et al., 2023) suggested that the percentages of HLA‐DR CD45RA‐Tregs and CD28‐Treg‐like cells significantly increased with age and can serve as immunologic markers of reproductive aging. Additionally, Li et al. (Li et al., 2022) revealed that aging altered immune cell responses, especially by toning down Th17 cells, counteracting experimental autoimmune uveitis challenge in old mice. Beyond CD4^+^ T cells, other immune cell populations, including B cells (Camell et al., 2019), NK cells (Tang et al., 2022), and neutrophils (Martínez‐Alberquilla et al., 2022), have also been implicated in the development of aging and related diseases. Therefore, considering the heterogeneity and functional complexity of immune cells, further studies on the effects of miR‐7 on the function of other immune cells, including the functional subsets of CD4^+^T cells, and its potential role in the aging process will not only deepen our understanding of the regulatory mechanisms of miR‐7 in aging, but also clarify the mechanisms of immune‐mediated aging.

Interleukin‐1β (IL‐1β), a pro‐inflammatory factor primarily produced by immune cells (such as macrophages) (Lopez‐Castejon & Brough, 2011; Zhang et al., 2022), has been implicated in the aging process of various cells and tissues, as well as the occurrence and development of age‐related diseases (Denaës et al., 2016; Isorce et al., 2016). Notably, our study revealed a significant down‐regulation of IL‐1β levels in liver tissue upon miR‐7 deficiency, coinciding with a remarkable slowdown of the aging process in model mice. Moreover, in vivo neutralization of IL‐1β significantly improved the aging process in the model mice. These results suggest that regulation of IL‐1β by miR‐7 plays a crucial role in the aging process of d‐gal‐induced model mice. Our previous research also demonstrated that miR‐7 deficiency significantly improved the lung pathology of acute lung injury in mice, accompanied by a significant down‐regulation of IL‐1β expression (Zhao et al., 2016). Conversely, in brain tissue inflammation, miR‐7 deficiency exacerbated related pathology and up‐regulated IL‐1β expression (Yue et al., 2020), indicating the complex regulatory mechanisms of miR‐7 on IL‐1β production. Furthermore, our results suggest that miR‐7 regulates IL‐1β production in Kupffer cells by targeting KLF4, independent of the Nlrp3 inflammasome. This suggests the existence of other secretory pathways by which miR‐7 targeting KLF4 regulates IL‐1β, which warrants further exploration. Finally, it should not be ignored that our data showed that the expression levels of various aging‐related inflammatory factors, including IL‐6, in the liver, lung, and kidney tissues of aging mice were down‐regulated to varying degrees after the deficiency of miR‐7. While these cytokines were not further explored in this study, given the intricate role of inflammatory factors in aging, the regulatory role of miR‐7 on their expression and the complex potential links among them still need to be further studied.

Kupffer cells are liver‐residing self‐renewing macrophages that lead to the production of cytokine and chemokine through activation of signaling pathways such as NF‐κB. They play an important role in chronic alcoholic liver disease, non‐alcoholic steatohepatitis, and viral hepatitis (Hellerbrand et al., 1999; Slevin et al., 2020; Tuttle et al., 2020). Additionally, Kupffer cells are involved in the production of reactive oxygen species (ROS) (Wilkinson et al., 2019), contributing to oxidative stress and inflammation, both hallmarks of aging. It is more important to note that the accumulation of focal or tissue‐specific senescent cells in any tissue is a driver of aging in organisms (Sindrilaru et al., 2011). Some studies have found that in certain cases, the macrophage phenotype dependent on the environment causes senescence of surrounding cells (Rampelli et al., 2020; Yu et al., 2023). Importantly, macrophage senescence can contribute to the progression of aging‐related diseases (Nie et al., 2023; Zheng et al., 2023). For example, senescent macrophages release pro‐inflammatory cytokines, chemokines, and ROS, resulting in cardiovascular inflammation (Sadhu et al., 2021). The senescent macrophages also contribute to plaque growth and rupture, impacting the development of atherosclerosis (Childs et al., 2016). Additionally, senescent macrophages with impaired cholesterol efflux can promote the onset of age‐related macular degeneration (Sene et al., 2013). Our results also demonstrate that miR‐7 influences the aggregation of senescent Kupffer cells in the murine liver and regulates the secretion of IL‐1β, thereby affecting liver aging. This suggests that the instability of gene expression may be a key factor driving the aggregation of specific senescent cells in tissues, thus affecting the aging process. Recent studies have also shown that aging of different organs not only exhibits temporal and spatial heterogeneity, but also has a certain cross‐talk relationship (Crislip et al., 2022). Interestingly, our study observed that the liver, lung, and kidney showed different degrees of aging phenotype after adoptive transfer of miR‐7‐deficient CD4^+^T cells and neutralization of IL‐1β in vivo. These findings suggest that the regulatory effects of miR‐7 on aging in different organs are heterogeneous. Therefore, successive research works on the distinct roles of miR‐7 in these different organs are valuable insights into the exploration on the exact role of miR‐7 in aging process and the development of intervention strategies against aging in the future.

KLF4 is an evolutionarily conserved zinc finger‐containing transcription factor that regulates diverse cellular processes such as cell growth, proliferation, and senescence, by activating the NF‐κB signaling pathways (Blacher et al., 2022; Ghaleb & Yang, 2017); it is widely recognized for its importance in organ development and disease. Accumulating evidence has suggests that KLF4 signaling is central to the regulation of aging processes, as well as the response to tissue injury and inflammation (Blacher et al., 2022; Mosteiro et al., 2016). Just as mentioned above, we found that KLF4 expression decreased in the liver tissue in aged mice. Of note, suppressing KLF4 expression reversed the effect of miR‐7 deficiency on senescent macrophages, accompanied by a significant up‐regulation of p16^INK4A^ and p21^CIP1^ expression and increased IL‐1β production. Furthermore, KLF4 and miR‐7 were predominantly co‐localized in Kupffer cells rather than hepatic cells in aging mice. Notably, in the absence of miR‐7, inhibition of KLF4 expression promoted the production of pro‐inflammatory cytokine IL‐1β by senescent Kupffer cells in response to d‐gal, which was closely correlated with the altered activation of the NF‐κB signaling pathway. Hence, these data demonstrate the important role of the miR‐7/KLF4 axis in Kupffer cell senescence in aging mice. In addition, other factors, such as ATG7 and SIRT1, which are regulated by miR‐7, may play an important role in the process of Kupffer cell senescence. For example, Yang et al. (Yang et al., 2016) demonstrated that ATG7, a core gene associated with autophagy, is significantly reduced in aged Kupffer cells compared to their younger counterparts. SIRT1, a key polymodulin of the third class of histone deacetylase, is involved in age‐related multiple transcription and protein target regulation and is lowly expressed in the aging liver (Hunt et al., 2019). Interestingly, we also observed up‐regulation of these factors in liver tissue in the absence of miR‐7. Additionally, blocking the KLF4 did not completely nullify the effect of miR‐7 in Kupffer cells, indicating that these factors, which were not explored in the present study, might also contribute to the effect of miR‐7 on Kupffer cell senescence.

In conclusion, this study not only demonstrates that miR‐7 deficiency can slow down the aging process of d‐gal‐induced model mice, but also reveals that miR‐7 regulates the IL‐1β secretion in senescent Kupffer cells by modulating the KLF4/NF‐κB pathway, thereby affecting the aging of d‐gal‐induced model mice. This discovery provides an important theoretical and scientific foundation for further research on the development of new targeted gene therapy strategies for anti‐aging and related diseases.

## AUTHOR CONTRIBUTIONS

J.Z. and L.X. designed the study. Y.W., H.Q., S.C., D.L., X.Z., and M.G. performed the experiments. Y.W., C.C., M.Q., and Y.Z. analyzed the data. Y.W., D.X., J.Z., and L.X. wrote the manuscript with contribution from all coauthors.

## CONFLICT OF INTEREST STATEMENT

All authors declare that the research was conducted in the absence of any commercial or financial relationships that could be construed as a potential conflict of interest.

## Supporting information


Appendix S1

## Data Availability

The authors declare that there are no primary datasets and computer codes associated with this study. All data and materials are available to the researchers once published.
