# Supplementary material for: MicroRNA‐7 deficiency ameliorates d‐galactose‐induced aging in mice by regulating senescence of Kupffer cells
Source: Aging Cell. 2024 Mar 17;23(6):e14145. doi: 10.1111/acel.14145 (PMC11166366; doi:10.1111/acel.14145)
Supplement: Supplementary file 1 — Appendix S1 [file ACEL-23-e14145-s001.pdf]

MicroRNA-7 deficiency ameliorates D-galactose-induced aging in mice by regulating senescence of Kupffer cells

Ya Wang[1,2#], Hui Qiu[1,2#], Shipeng Chen[1,2], Dongmei Li[1,2], Xu Zhao[1,4], Mengmeng Guo[1,2], Nana Li[2], Chao Chen[1,2], Ming Qin[1,2], Ya Zhou[3], Daimin Xiao[5*], Juanjuan Zhao[1,2*], Lin Xu[1,2*]

[1] Department of Immunology, Zunyi Medical University, Guizhou, 563000 China

[2] Key Laboratory of Gene Detection and Treatment of Guizhou province, Guizhou Zunyi, 563000 China;

[3] Department of Medical physics, Zunyi Medical University, Guizhou, 563000 China

[4] School of Medicine, Guizhou University, Guiyang 550025, Guizhou, China.

[5] Kweichow Moutai Hospital, Guizhou Renhuai, 564501 China

[#] These authors contributed equally to this work.

[*] **Correspondence to:**

Dr. Lin Xu, Department of immunology, Zunyi Medical University, GuiZhou Province, 563003 P.R.China, Email: xulinzhouya@163.com

Dr. Juanjuan Zhao, Department of immunology, Zunyi Medical University, GuiZhou Province, 563003 P.R.China, Email: jj.z.2008@163.com

Dr. Daimin Xiao, Kweichow Moutai Hospital, Guizhou Renhuai, 564501 P.R.China, Email: 372422022@qq.com

Number of Figures: 6

Number of supplementary Figures: 10

Number of supplementary Tables: 1

Supplementary Figure 1-10

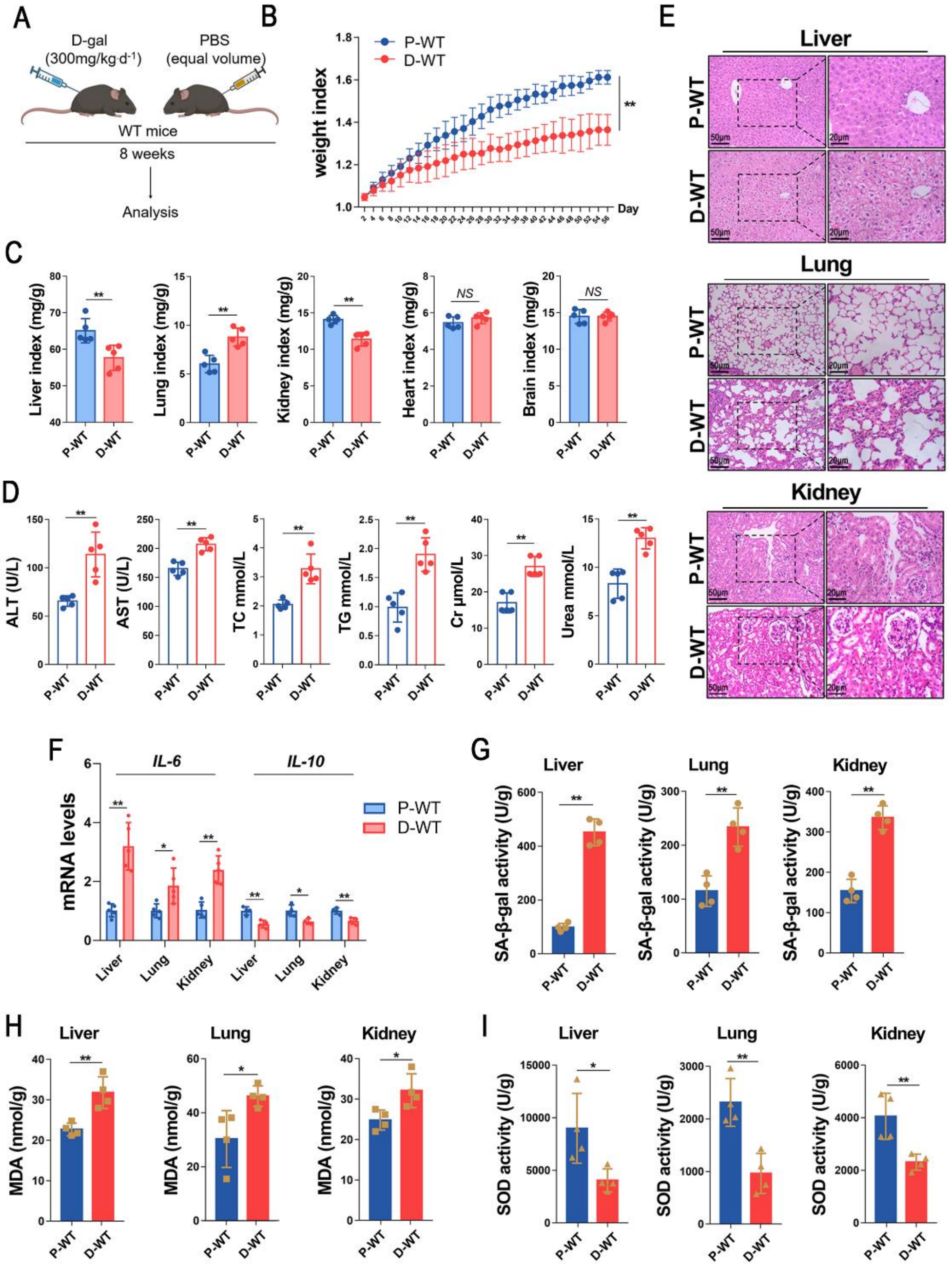

Supplementary Figure 1. Establishment of the D-gal induced murine aging model. (A) Schematic representation of the animal experiments for D-gal induced murine aging model. WT mice (6-7 weeks old, n=5) were given 300mg/kg·d^{-1} D-gal solution or equal volume PBS for 8weeks; the mice were sacrificed on the 56th day. The weight index (B) and organ index (C) of WT mice after D-gal treatment were observed. The concentration of serum ALT, AST, TC, TG, Cr and Urea were detected and analyzed (D). Histopathology of liver, lung, and kidney tissues was performed by H&E staining (left, 50μm; right, 20μm) (E). The relative expression levels of cytokines *IL-6* and *IL-10* in the liver, lung, and kidney tissues were assessed using Real-time PCR assay (F). The activity of SA-β-gal (G) and SOD (I), the content of MDA (H) in the liver, lung, and kidney were detected using biochemical reagent kit. The values are the means ± SD (n=5). **P<0.05*, ***P<0.01*.

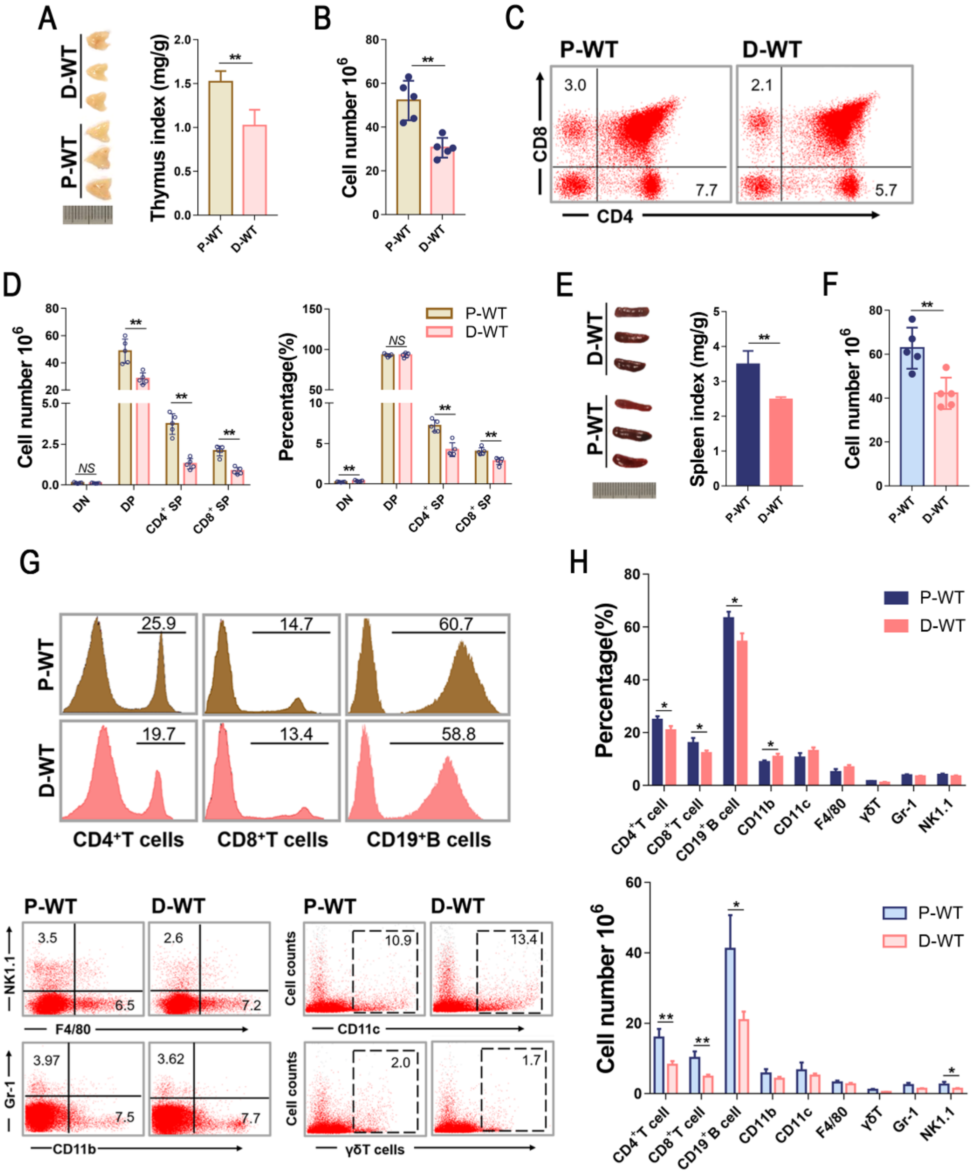

Supplementary Figure 2. Changes of immune organs and immune cells in aging mice. WT mice (6-7 weeks old, n=5) were given 300mg/kg·d^{-1} D-gal solution or equal volume PBS for 8weeks; The size, organ index (A) and cell numbers (B) of thymus were assessed. The percentage and cell numbers of DN cells, DP cells, CD4^{+} SP cells, CD8^{+} SP cells in thymus were detected from using FCM assay and calculated(C-D). The size, organ index (E) and cell numbers (F) of spleen were measured. The percentage and cell numbers of CD4^{+} T cells, CD8^{+} T cells, CD19^{+} B cells, NK1.1, CD11c, CD11b, Gr-1, F4/80, and γδT cells in splenocytes were detected by FCM assay and calculated respectively (G-H). The values are the means ± SD (n=5). *$P<0.05$, **$P<0.01$.

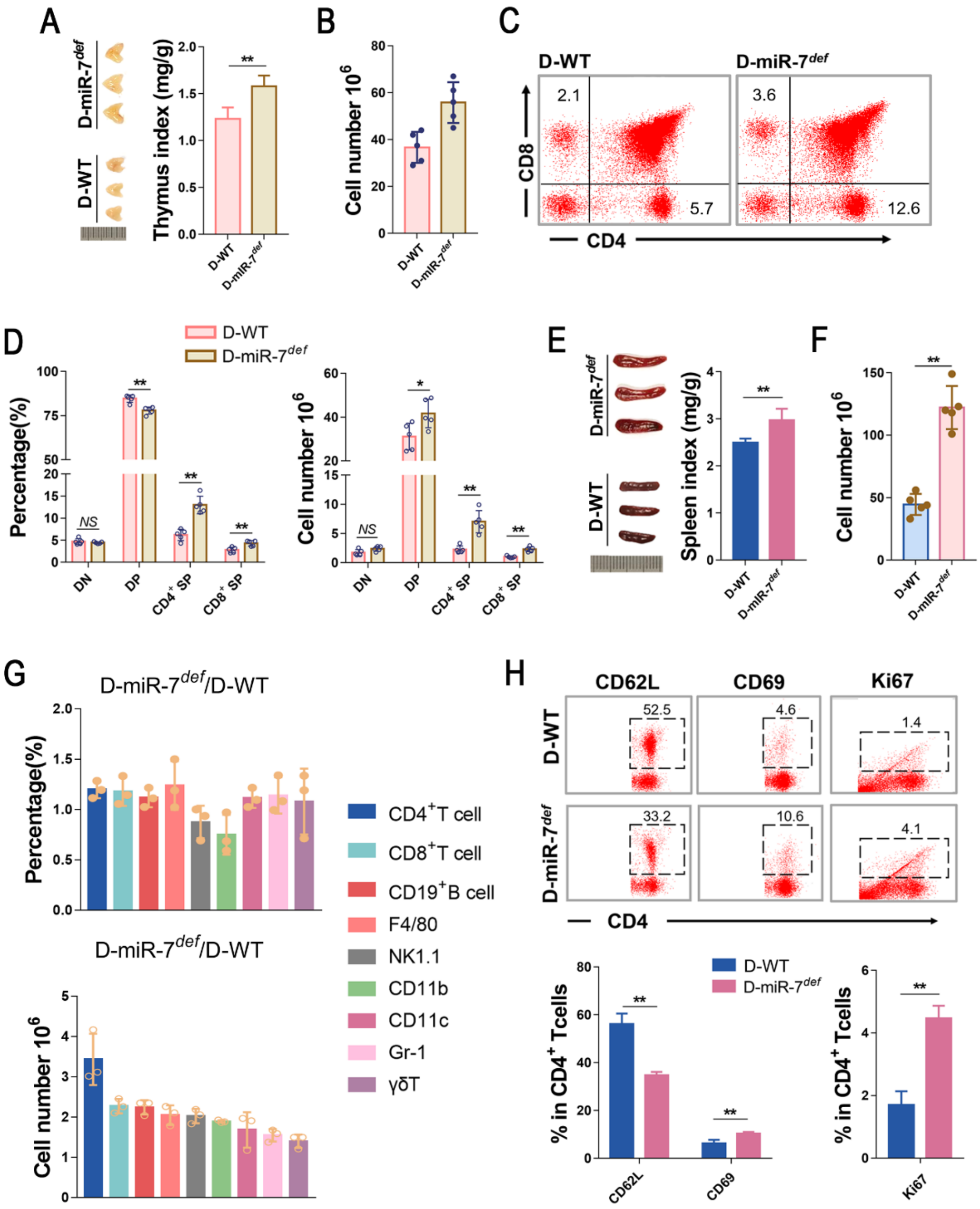

Supplementary Figure 3. Effects of miR-7 deficiency on immune organs and immune cells in aging mice. MiR-7^{def} mice and WT mice (6-7 weeks old, n=5) were given 300mg/kg·d^{-1} D-gal solution for 8 weeks, and the mice were sacrificed on the 56th day. The size, organ index (A) and cell numbers (B) of thymus were measured. The percentage and cell numbers of DN cells, DP cells, CD4^{+} SP cells, CD8^{+} SP cells in thymus were detected by FCM assay and calculated respectively(C-D). The size, organ index (E) and cell numbers of spleen (F) were assessed. The percentage and cell numbers of CD4^{+} T cells, CD8^{+} T cells, CD19^{+} B cells, NK1.1, CD11c, CD11b, Gr-1, F4/80, and γδ T cells in splenocytes were detected by FCM assay and calculated respectively (G). The percentage of CD4^{+} CD62L^{+}T cells, CD4^{+} CD69^{+} T cells, and CD4^{+} Ki67^{+} T cells in splenocytes were detected by FCM and calculated respectively (H). The values are the means ± SD (n=5). *$^{*}P<0.05$, $^{**}P<0.01$.*

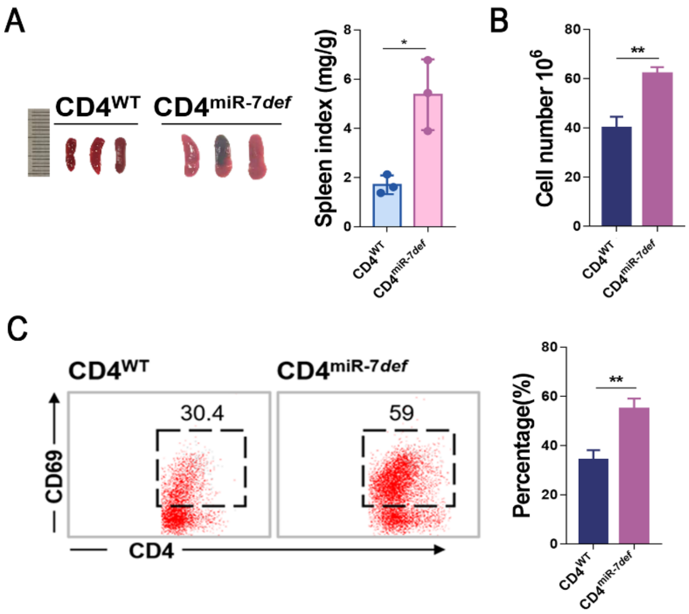

Supplementary Figure 4. Transferred miR-7 deficient CD4$^+$T cells promote splenic CD4$^+$T cell activation. CD4$^+$CD62L$^+$T cells purified by MACS in splenocytes from WT and miR-7^{def} mice (6-7 weeks, n=3) were transferred into syngeneic Rag1$^{-/-}$ mice through tail vein injection every 2 weeks, and these mice were administered 300 mg/kg D-gal (i.h.) for 8 weeks, and these mice were sacrificed on the 56th day (Figure 2B). The size and organ index (A) and cell numbers of spleen (B) were detected. The expression of activation marker CD69 on CD4$^+$ T cells in splenocytes was detected using FCM assay and calculated respectively(C). The values are the means ± SD (n=5). **P<0.05, **P<0.01.*

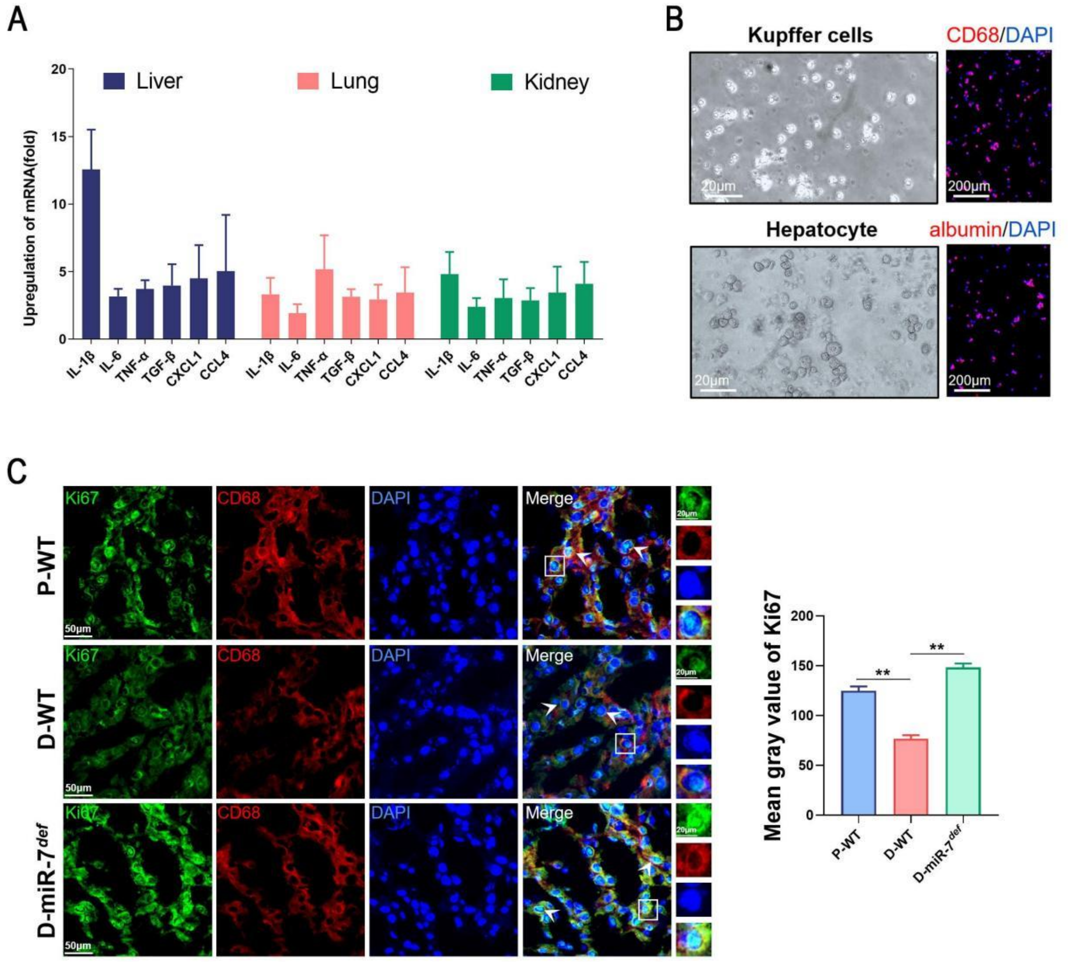

Supplementary Figure 5. MiR-7 deficiency promotes the proliferation of Kupffer cells in aging mice. (A) The relative expression levels of inflammatory cytokines *IL-1β*, *IL-6*, *TNF-α*, *TGF-β*, *CXCL1*, and *CCL4* were assessed in the liver, lung and kidney tissue by Real-time PCR assay. The multiples of their up-regulation were then analyzed in D-WT group; (B) Hepatocytes (characteristic marker albumin) and Kupffer cells (characteristic marker CD68) were isolated from the liver of mice and identified by Immunofluorescence. (C) Immunofluorescence results for Ki67 and CD68 in the liver from P-WT, D-WT, and D-miR-7^{def} mice. The values are the means ± SD (n=3),*$P<0.05$, **$P<0.01$.

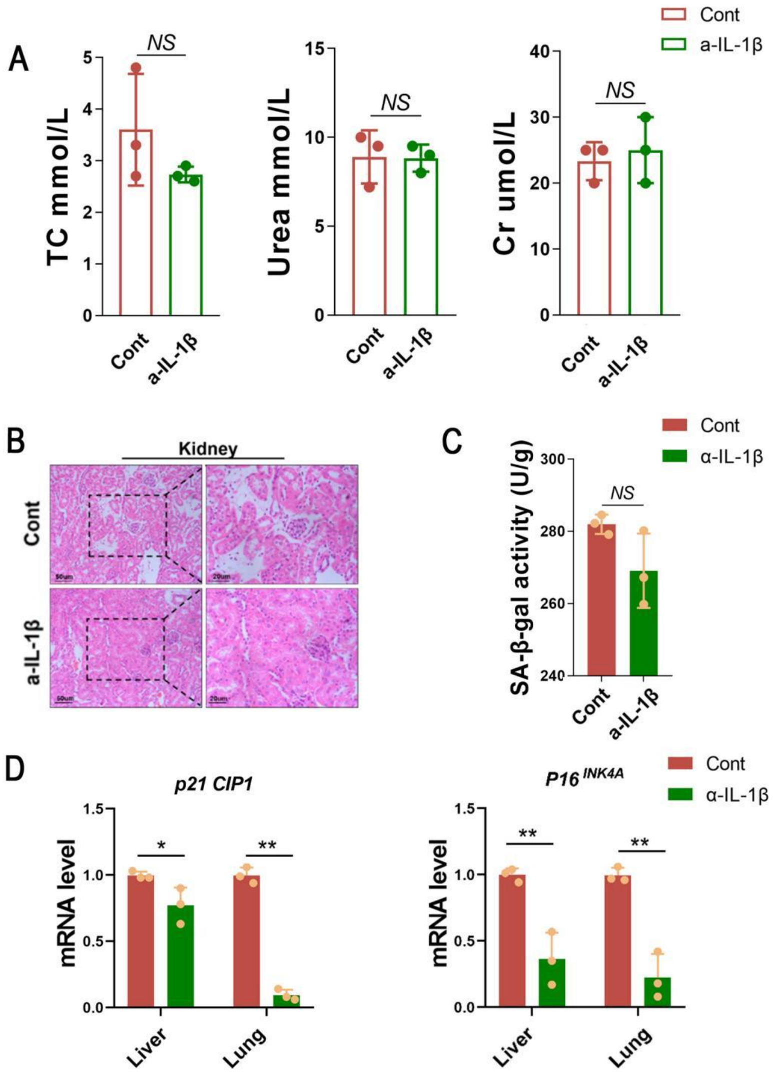

Supplementary Figure 6. Neutralizing IL-1β *in vivo* can improve murine aging induced by D-gal. (A) The concentration of the serum TC, Urea and Cr were detected and analyzed. (B) Histopathology of kidney was performed by HE staining (left, 50μm; right, 20μm). (C) The activity of SA-β-gal in Kidney tissue was detected by a biochemical reagent kit. (D) The relative expression levels of senescence markers $p21^{CIP1}$and $p16^{INK4A}$ were assessed in the liver and lung tissue by Real-time PCR assay in Control (Cont) group and anti-IL-1β (aIL-1β) group. The values are the means ± SD (n=5). **P*< 0.05, ***P*< 0.01.

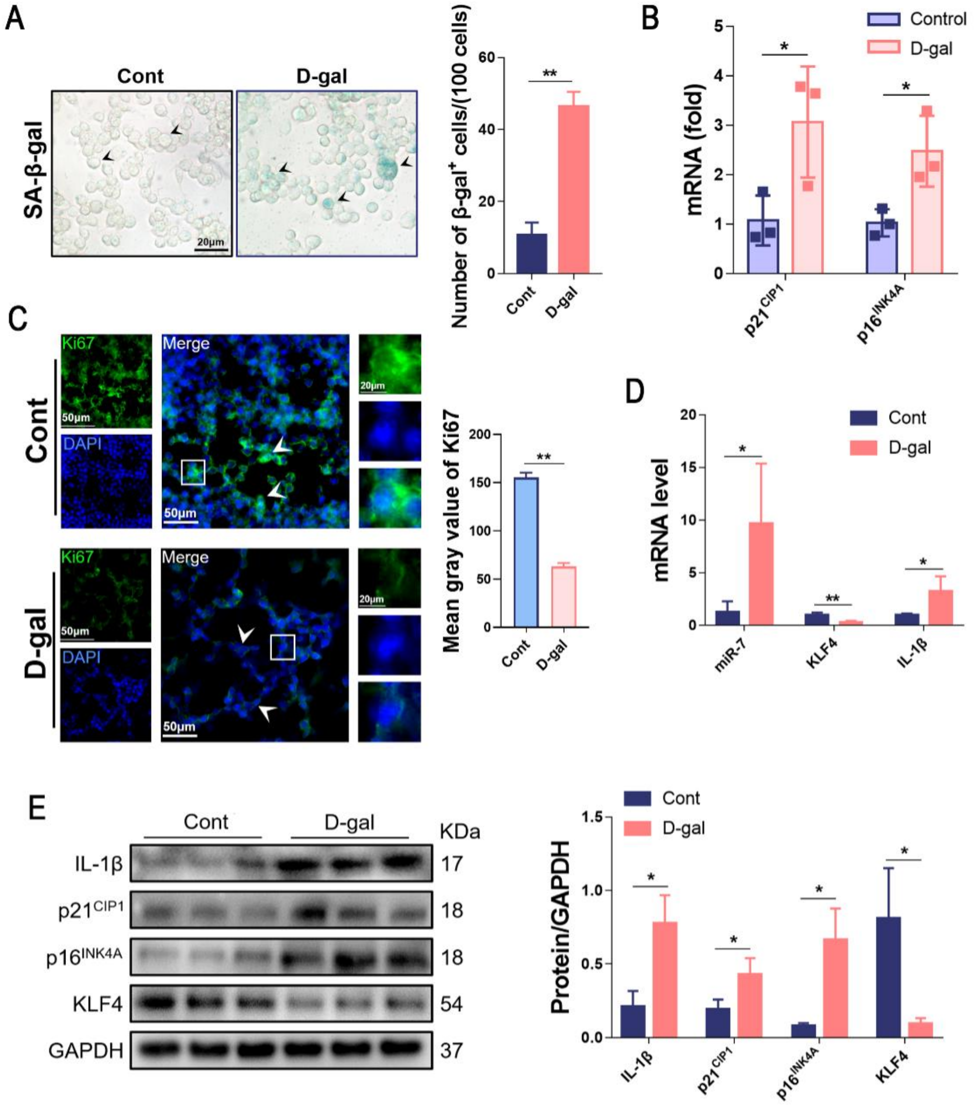

Supplementary Figure 7. RAW264.7 cell senescence was induced by D-gal *in vitro*. (A) SA-β-gal staining analyzed the number of β-gal positive cells in RAW264.7 cells with or without 10g/L D-gal. The total number of SA-β-gal positive cells in 100 randomly selected cells was observed by optical microscopy. (B) The mRNA level of $p16^{INK4A}$ and $p21^{CIP1}$ in RAW264.7 cells with or without 10g/L D-gal was examined by Real-time PCR assay. (C) Immunofluorescence for Ki67 expression in RAW264.7 cells. (D) The mRNA level of *miR-7*, *KLF4* and *IL-1β* in RAW264.7 cells with or without 10g/L D-gal was examined by Real-time PCR. (E) The protein expression levels of $p16^{INK4A}$, $p21^{CIP1}$, KLF4, and IL-1β were detected by western blot assay and calculated. The values are the means ± SD (n=3), $*P < 0.05$, $**P < 0.01$.

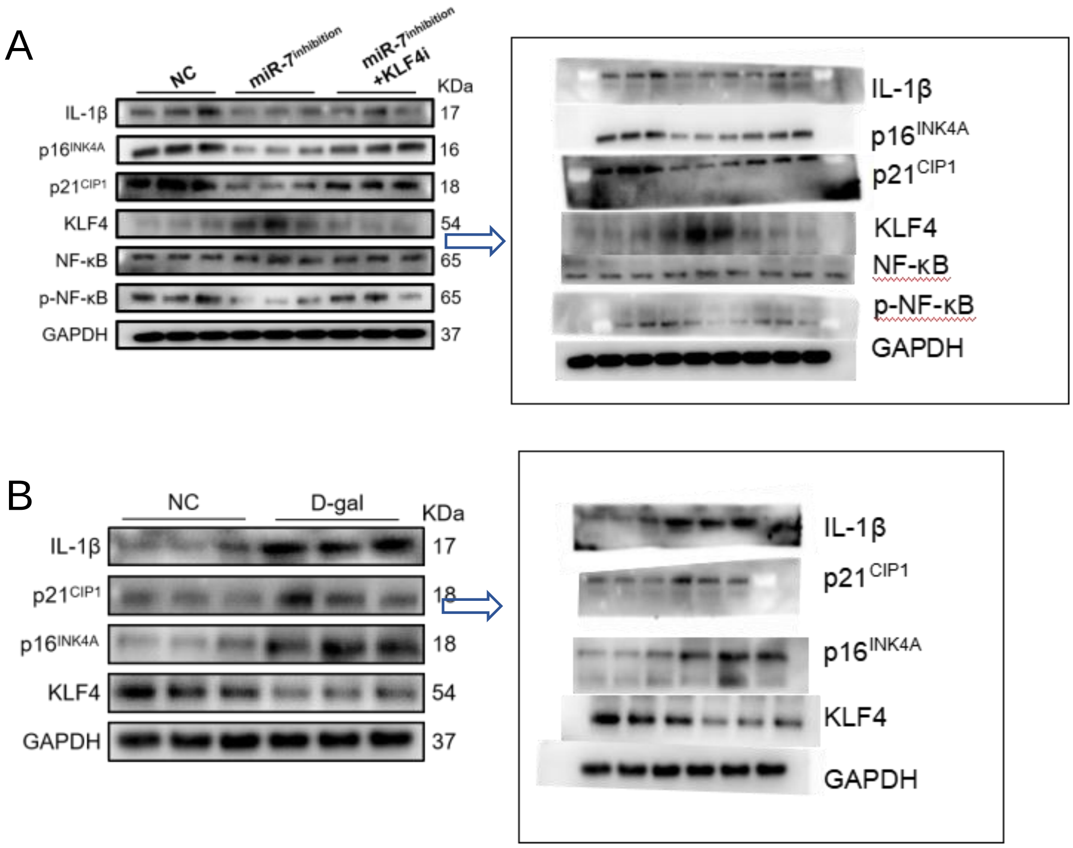

Supplementary Figure 8. Uncropped immunoblots. (A) Uncropped immunoblots for Supplementary Figure 6F; (B) Uncropped immunoblots for Figure S7E.

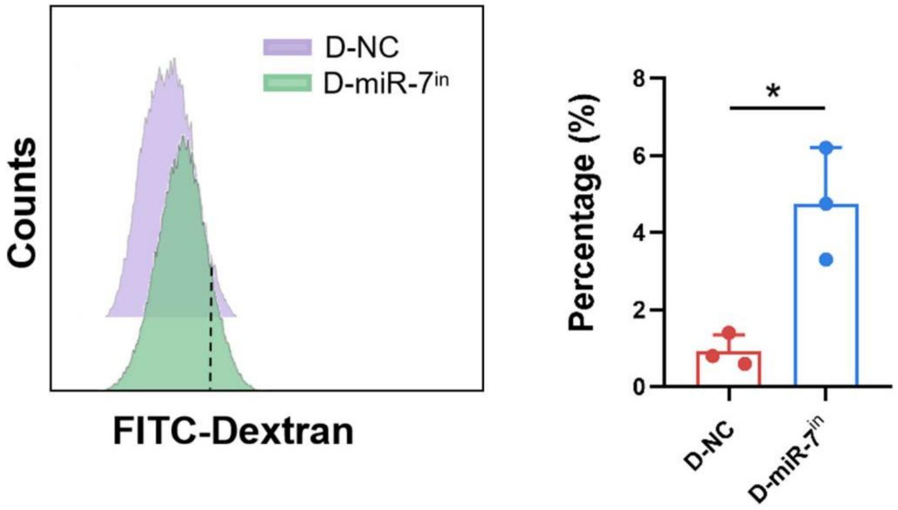

Supplementary Figure 9. MiR-7 deficiency enhances the phagocytic function of senescent macrophages. Murine macrophages RAW264.7 cells were treated with NC/miR-7inhibitors (100 nM), 24 h later, these cells were continuously treated with D-gal for 48 h. The phagocytic functions of senescent macrophages were detected. The values are the means ± SD (n=3), $*P < 0.05$, $**P < 0.01$.

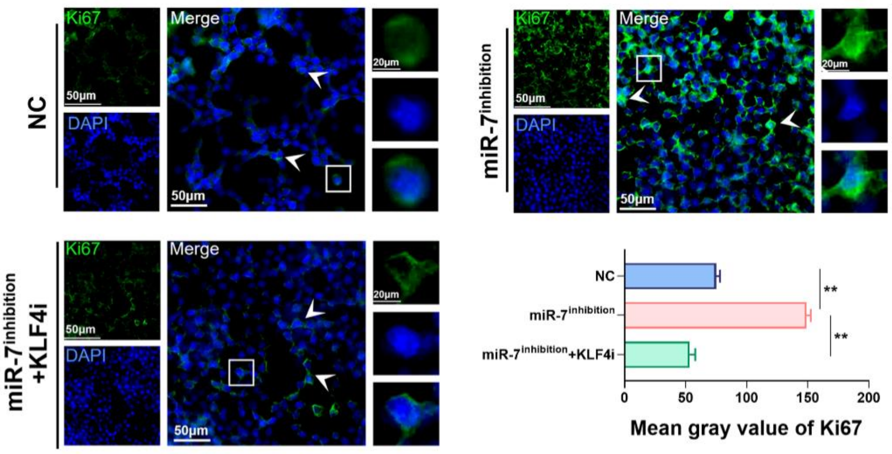

Supplementary Figure 10. MiR-7 controls the proliferation of senescent macrophages via KLF4 *in vitro*. Murine macrophages RAW264.7 cells were treated with NC/miR-7inhibitors/miR-7 inhibitors+KLF4-RNAi (100 nM), 24 h later, these cells were continuously treated with D-gal for 48 h. Immunofluorescence for Ki67 expression in RAW264.7 cells with D-gal treatment of each group. The values are the means ± SD (n=3), $^{*}P < 0.05$, $^{**}P < 0.01$.

Supplementary Table 1. Primers for Real-time PCR in this study

Gene	Forward primer 5'-3'	Reverse primer 5'-3'
IL-6	GGAAATCGTGGAAATGAG	AGGACTCTGGCTTTGTCT
IL-10	TACAGCCGGGAAGACAATAA	AGGAGTCGGTTAGCAGTATG
TNF-α	CAGGGGCCACCACGCTCTTC	TTTGTGAGTGTGAGGGTCTGG
TGF-β1	GGCGGTGCTCGCTTTGTA	TCCCGAATGTCTGACGTATTGA
CXCL1	CACCCGCTCGCTTCTCTG	TCTTGAGGTGAATCCCAGCC
CCL4	CCCAGCTCTGTGCAAACCTA	CCATTGGTGCTGAGAACCCT
IL-1β	GCAACTGTTCCTGAACTCAACT	ATCTTTTGGGGTCCGTCAACT
$P21^{CIP1}$	AGTCCTTTGCCCTGAACTGC	GCGGATCTTGAGGGTGAAAT
$P16^{INK4A}$	CGAACTCGAGGAGAGCCATC	TACGTGAACGTTGCCCATCA
KLF4	GCCCAACACACACGACTTC	GGCAGGAAAGGAGGGTAGTT
ATG7	GCCAACTCCACACTGCTTTC	TCTTCTGGGTCAGTTCGTGC
SIRT1	GATGACAGAACGTCACACGC	ACAATCTGCCACAGCGTCAT
FoxO6	CAGGAGTAGCCGAGGGTTCC	AGCGGACCATCCAGTCGTAG
YY1	TGGCATTGACCTCTCAGACC	GCTCTCAACGAACGCTTTGC
NIrp3	CAGAGCCTACAGTTGGGTGAAATG	CACGCCTACCAGGAAATCTCG
GAPDH	AGAAGGCTGGGGCTCATTTG	AGGGGCCATCCACAGTTCTTC
